# The role of Cytochrome b_6_f in the control of steady-state photosynthesis: a conceptual and quantitative model

**DOI:** 10.1007/s11120-021-00840-4

**Published:** 2021-05-17

**Authors:** J. E. Johnson, J. A. Berry

**Affiliations:** Dept. Global Ecology, Carnegie Institution, Stanford, CA 94305 USA

**Keywords:** Model, Electron transport, Photosystem II, Photosystem I, Cytochrome $\hbox {b}_{6}\hbox {f}$, Rubisco

## Abstract

Here, we present a conceptual and quantitative model to describe the role of the Cytochrome $\hbox {b}_{6}\hbox {f}$ complex in controlling steady-state electron transport in $\hbox {C}_{3}$ leaves. The model is based on new experimental methods to diagnose the maximum activity of Cyt $\hbox {b}_{6}\hbox {f}$ in vivo, and to identify conditions under which photosynthetic control of Cyt $\hbox {b}_{6}\hbox {f}$ is active or relaxed. With these approaches, we demonstrate that Cyt $\hbox {b}_{6}\hbox {f}$ controls the trade-off between the speed and efficiency of electron transport under limiting light, and functions as a metabolic switch that transfers control to carbon metabolism under saturating light. We also present evidence that the onset of photosynthetic control of Cyt $\hbox {b}_{6}\hbox {f}$ occurs within milliseconds of exposure to saturating light, much more quickly than the induction of non-photochemical quenching. We propose that photosynthetic control is the primary means of photoprotection and functions to manage excitation pressure, whereas non-photochemical quenching functions to manage excitation balance. We use these findings to extend the Farquhar et al. (Planta 149:78–90, 1980) model of $\hbox {C}_{3}$ photosynthesis to include a mechanistic description of the electron transport system. This framework relates the light captured by PS I and PS II to the energy and mass fluxes linking the photoacts with Cyt $\hbox {b}_{6}\hbox {f}$, the ATP synthase, and Rubisco. It enables quantitative interpretation of pulse-amplitude modulated fluorometry and gas-exchange measurements, providing a new basis for analyzing how the electron transport system coordinates the supply of Fd, NADPH, and ATP with the dynamic demands of carbon metabolism, how efficient use of light is achieved under limiting light, and how photoprotection is achieved under saturating light. The model is designed to support forward as well as inverse applications. It can either be used in a stand-alone mode at the leaf-level or coupled to other models that resolve finer-scale or coarser-scale phenomena.

## Introduction

### Overview

At present, a large number of measurement techniques can be brought to bear on studying terrestrial photosynthesis at and above the leaf-level. Most measurement techniques target one of two broad categories of phenomena: how leaves absorb, emit, and scatter light or how leaves produce and consume gases. While it is possible to interpret both categories of measurements with quantitative models of photosynthesis, quantitative interpretations of gas-exchange are currently much more common than quantitative interpretations of radiative fluxes. The premise of this paper is that developing a more quantitative interpretation of the radiative fluxes is the key to building more complete understanding of how photosynthesis works at the leaf-level, as well as more accurate strategies for quantifying photosynthesis at the canopy-level.

Toward this end, our point of departure is the quantitative framework that is most widely used for studying photosynthesis at and above the leaf-level: the model of $\hbox {C}_{3}$ photosynthesis by Farquhar et al. (1980). To date, the Farquhar et al. (1980) model has provided a strong foundation for interpreting and simulating the gas-exchange fluxes that are associated with photosynthesis because it is grounded in a mechanistic representation of carbon metabolism. However, it has also provided a comparatively weak foundation for interpreting and simulating the radiative fluxes that are associated with photosynthesis because it has relied on an empirical representation of electron transport. The aim of this paper is to introduce a new model of electron transport that is designed to replace the empirical scheme in the Farquhar et al. (1980) framework.

The Farquhar et al. (1980) model was originally designed to interpret leaf-level measurements of $\hbox {CO}_2$ assimilation under different light intensities, temperatures, and $\hbox {CO}_2$ and $\hbox {O}_2$ partial pressures. It has been used in a wide range of applications (e.g., see reviews by von Caemmerer 2000; Long and Bernacchi 2003; Sharkey et al. 2007; von Caemmerer et al. 2009; von Caemmerer 2013; Porcar-Castell et al. 2014; Rogers et al. 2017; Mohammed et al. 2019; von Caemmerer 2020). One frequent application has been to use the leaf-level model in a stand-alone form to infer the biochemical properties of leaves from gas-exchange measurements. Another frequent application has been to embed the leaf-level model in larger canopy models to predict land surface feedbacks on weather and climate. The reason that the model has been useful in such a breadth of applications is that it explains the environmental responses of photosynthetic gas-exchange in a way that is both accurate and simple.

Since the original Farquhar et al. (1980) model was published, there has been an expansion in the availability of optical measurements that probe photosynthesis (e.g., 650–850 nm fluorescence signals from PS II and PS I, 810–830 nm absorbance signal from PS I, 540–580 nm absorbance signals from Cyt $\hbox {b}_{6}\hbox {f}$, 500–540 nm absorbance signals related to the proton motive force). In parallel, there has also been an expansion in the availability of models describing the photosynthetic process (e.g., Laisk et al. 2009b; Yin et al. 2009; Yin and Struik 2009; Ebenhöh et al. 2011; Kuvykin et al. 2011; Zaks et al. 2012; Zhu et al. 2013; Ebenhöh et al. 2014; Tikhonov and Vershubskii 2014; Matuszyńska et al. 2016; Amarnath et al. 2016; Davis et al. 2017; Harbinson and Yin 2017; Bennett et al. 2018; Morales et al. 2018a, b; Bellasio 2019; Bellasio and Farquhar 2019; Gu et al. 2019; Matuszyńska et al. 2019; Herrmann et al. 2020). However, what has not yet emerged is a model that explains the environmental responses of optical signals in a way that is both accurate and simple.

We submit that this reflects the challenge of truly understanding how photosynthesis works as an integrated system. From this perspective, there are three major outstanding questions: (1) How does the electron transport system balance the supply of Fd, NADPH, and ATP to the dynamic demands of carbon metabolism? (2) How does the system maximize light-use efficiency under limiting light? (3) How does the system switch to a photoprotective mode under saturating light? We posit that the answers to all three questions center on Cyt $\hbox {b}_{6}\hbox {f}$. More than fifty years ago, in vitro studies demonstrated that the rate-limiting step in linear electron flow is mediated by Cyt $\hbox {b}_{6}\hbox {f}$ (Stiehl and Witt 1969), and that linear electron flow through Cyt $\hbox {b}_{6}\hbox {f}$ is subject to feedback control based on the excitation balance of PS II and PS I (Murata 1969) as well as the activity of carbon metabolism (West and Wiskich 1968). However, the connections between these three observations and their implications for the overall functioning of photosynthesis in vivo are still not fully understood (e.g., Haehnel 1984; Foyer et al. 1990; Genty and Harbinson 1996; Baker et al. 2007; Foyer et al. 2012; Johnson et al. 2014; Schöttler and Tóth 2014; Finazzi et al. 2016; Tikhonov 2018).

**Fig. 1:**
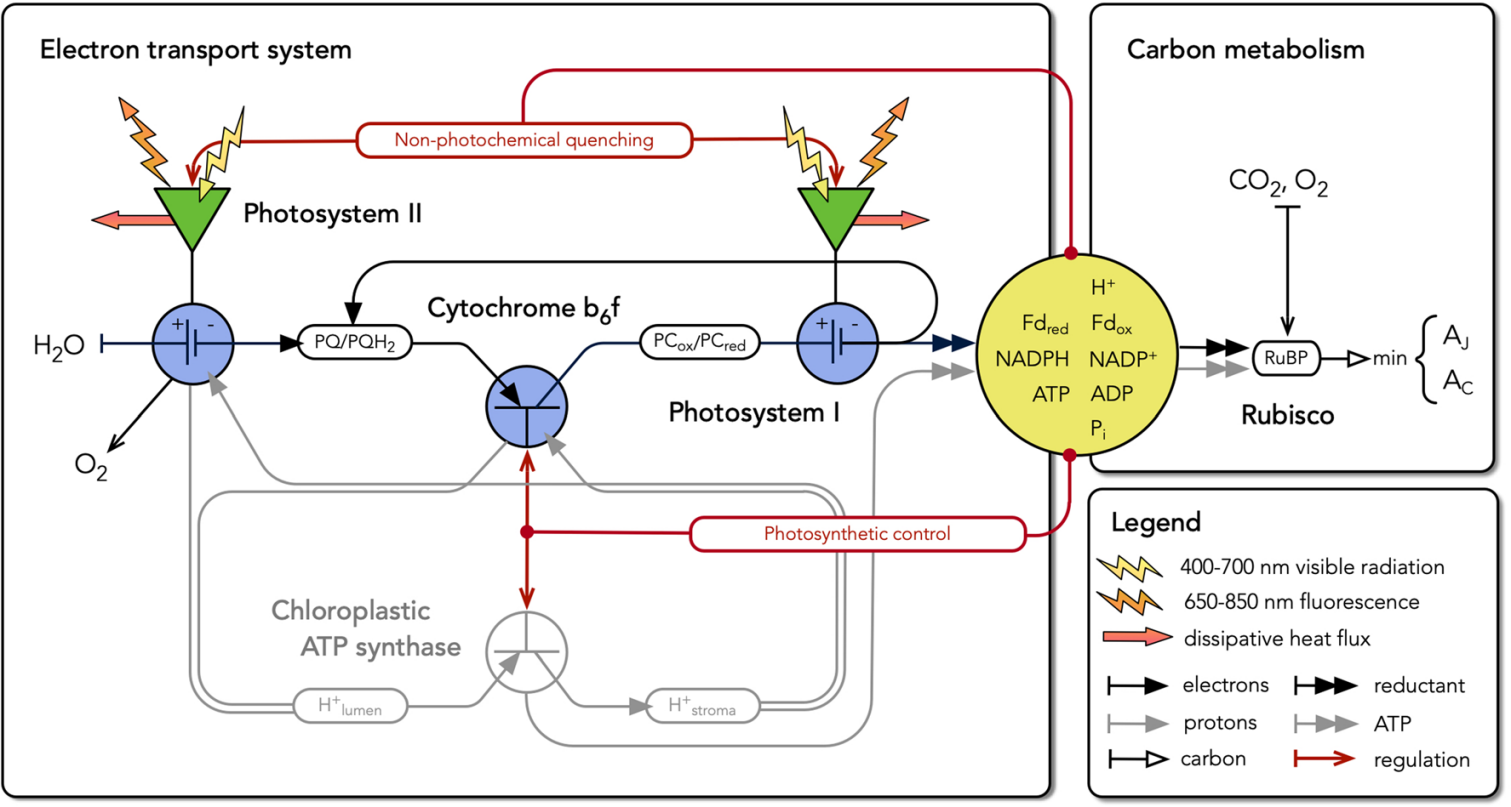
Electron transport system as an electrical circuit. In this model, we conceptualize Cyt $\hbox {b}_{6}\hbox {f}$ as a transistor, i.e., a regulated circuit element that uses variable conductance to control current flow. The linear flow of electrons from water to reductant is viewed as a light-driven current that is under the control of a hierarchy of regulatory feedbacks stemming from carbon metabolism. In limiting light, Cyt $\hbox {b}_{6}\hbox {f}$ presents maximal conductance to flow, and feedback from carbon metabolism adjusts the excitation of PS I and PS II in such a way as to balance the relative rates of linear and cyclic electron flow to the NADPH, Fd, and ATP requirements of the sinks. When light becomes saturating, feedback from carbon metabolism also decreases the apparent conductance of Cyt $\hbox {b}_{6}\hbox {f}$, controlling the linear flow of electrons through the plastoquinone pool and the associated flow of protons into the thylakoid lumen. In this way, the regulation of Cyt $\hbox {b}_{6}\hbox {f}$ simultaneously permits efficient photosynthesis and protects the system from photodamage. By expressing these concepts quantitatively, this model is able to simulate the steady-state gas-exchange and fluorescence fluxes that are associated with photosynthesis over the range of conditions experienced by leaves in nature

In this paper, we develop a conceptual and quantitative model that describes the role of Cyt $\hbox {b}_{6}\hbox {f}$ in controlling steady-state photosynthesis (Fig. 1). The model is based on experimental studies which introduce a procedure to estimate the maximum activity of Cyt $\hbox {b}_{6}\hbox {f}$ in vivo, and to identify the conditions under which feedback control of Cyt $\hbox {b}_{6}\hbox {f}$ is active or relaxed. The experimental results suggest that Cyt $\hbox {b}_{6}\hbox {f}$ functions like a transistor in an electrical circuit, operating at constant and maximum conductance (or minimum resistance) under limiting light and switching to a variable and higher resistance (or lower conductance) under saturating light. We use the transistor analogy to replace the empirical description of electron transport in the Farquhar et al. (1980) model with a mechanistic description that is based on the properties of Cyt $\hbox {b}_{6}\hbox {f}$. This creates a simple and accurate framework for interpreting and predicting the dynamics of photosynthesis across a wide range of environmental conditions. We first present the experimental studies and then proceed to the model.

### Experiment

The Farquhar et al. (1980) model predicts that electron transport should remain closely coupled with carbon metabolism across different light regimes, such that under any given condition the overall rate of photosynthesis corresponds to the minimum of the potential rates of these processes taken separately. The key idea underlying this prediction is that the steady-state fluxes in the photosynthetic system are under the control of the rate-limiting step in either electron transport or carbon metabolism, and a metabolic ‘switch’ controls the transition between limitation by electron transport and carbon metabolism. Originally, neither the identity of the rate-limiting step in electron transport, nor the exact nature of the switching mechanism, were resolved. Instead, the potential rate of linear electron transport, *J*, was described with an empirical function relating absorbed light to the curvature of the light response ($\theta$) and the maximum rate of electron transport observed under saturating light and $\hbox {CO}_2$ ($J_{\max }$). Similarly, the switch was implemented with a ‘minimum of’ procedure, and the resulting discontinuity was smoothed with another empirical curvature parameter.

**Fig. 2:**
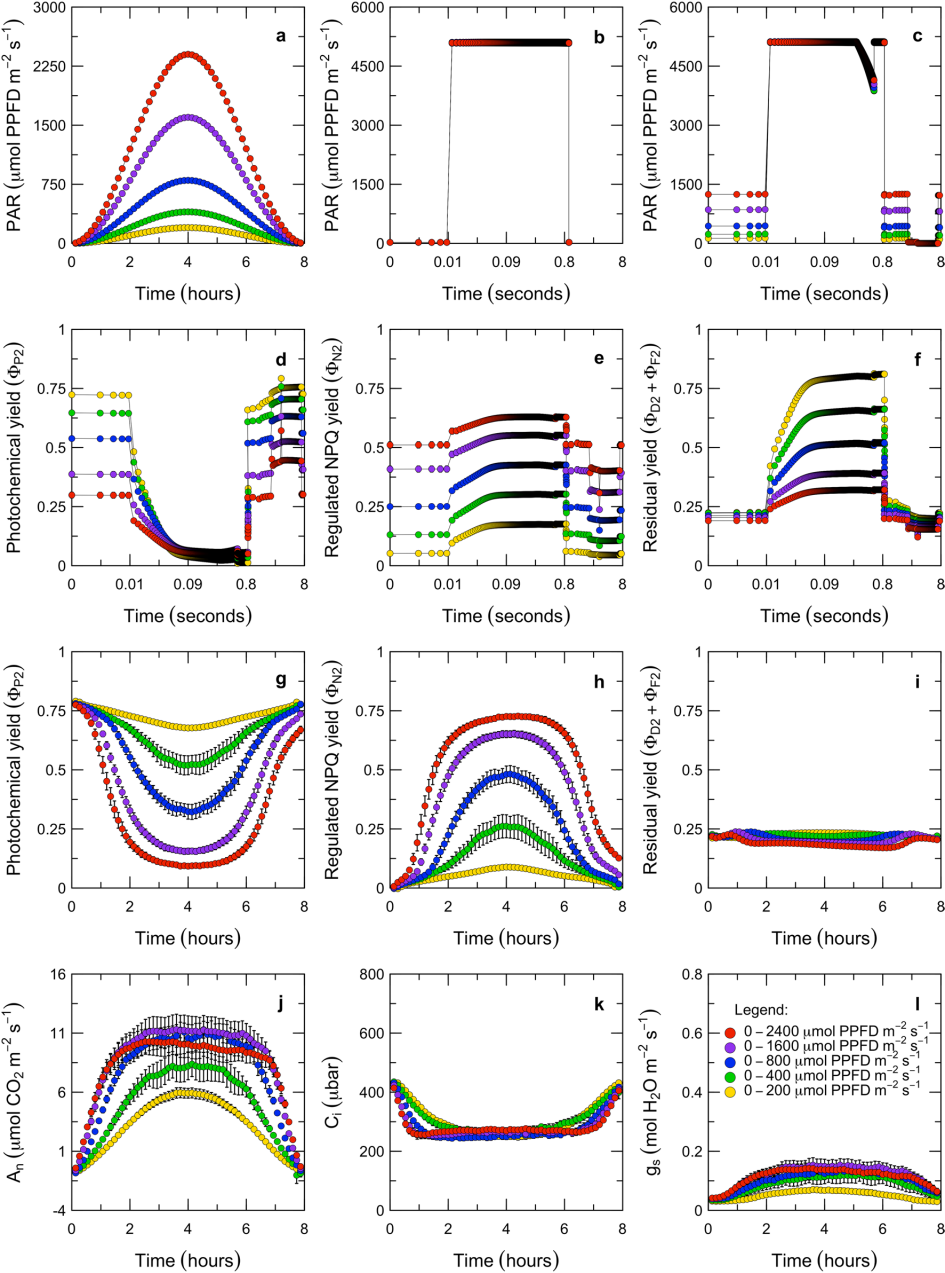
Response of *Populus fremontii* leaves to light sine waves. In this experiment, we varied the steady-state light intensity over the range of natural sunlight at different speeds and directions (**a**), and applied periodic saturating pulses at an intensity that was approximately double the maximum steady-state light intensity (**b**, **c**). We then characterized the transient fluorescence associated with each pulse (**d**–**f**), the steady-state fluorescence (**g**–**i**), and the steady-state gas-exchange (**j**–**l**). In (**a**, **g**–**l**), each point represents the mean of ${n} = 6$ replicates ± std. error, measured in 8 min increments over an 8 h period (*N* = 1830). In **b**–**f**, each point represents the mean of *n* = 343–354 replicates ± std. error, measured at 2 to 20 ms increments over each pulse ($N \approx$ 900,000). In **d**–**i**, the measured PS II yields are calculated as: $\varPhi _{P2} = 1 - F^{}_s/F^{'}_{m}$ (Genty et al. 1989); $\varPhi _{N2} = F^{}_{s} \cdot (1/F^{'}_{m} - 1/F^{}_{m})$ and $\varPhi _{D2}+\varPhi _{F2} = F^{}_{s}/F^{}_{m}$ (Hendrickson et al. 2004)

Here, we present an experiment that imposes transitions between light-limited and light-saturated conditions in a way that mimics a natural day, and explores the role of Cyt $\hbox {b}_{6}\hbox {f}$ in coordinating electron transport and carbon metabolism across these transitions (Fig. 2). We posit that the continuous curvature of the light response is caused by the kinetic restriction that Cyt $\hbox {b}_{6}\hbox {f}$ presents to electron flow through PS II and PS I, and that the potential capacity for electron transport is controlled by two regulated properties: the excitation balance of PS II and PS I and the maximum activity of Cyt $\hbox {b}_{6}\hbox {f}$. We further posit that the excitation balance of PS II and PS I is regulated by ‘non-photochemical quenching’ across the full range of light intensities, and that the switching behavior at the light saturation point corresponds to the onset of a feedback from carbon metabolism that is often referred to as ‘photosynthetic control’ of Cyt $\hbox {b}_{6}\hbox {f}$.

Two notes are needed about our use of this terminology. First, we will use the phrase ‘non-photochemical quenching’ (NPQ) to refer in a general way to the processes responsible for dissipation of excess excitation from the PS II antennae, and we will note explicitly when it is necessary to differentiate between different forms of this feedback (e.g., state transitions (qT), chloroplast movements (qM), psbS-dependent (qE) and zeaxanthin-dependent (qZ) quenching; Demmig-Adams et al. 2014). Second, ‘photosynthetic control’ has been defined in several different ways (e.g., Foyer et al. 2012), and we will use it to refer only to modulation of the apparent conductance of Cyt $\hbox {b}_{6}\hbox {f}$ to linear electron flow (LEF). This definition is intended to accommodate the current uncertainties as to the specific mechanisms of the feedback (e.g., Finazzi et al. 2016), and to emphasize that the functional effect of the feedback is restriction of the linear electron flux through Cyt $\hbox {b}_{6}\hbox {f}$.

#### Experimental design

This experiment was conducted with *Populus fremontii*, a broadleaf deciduous tree that is native to California and exhibits physiology that is typical of $\hbox {C}_{3}$ angiosperms. *P. fremontii* saplings were grown in a greenhouse in Stanford, California. During growth, the saplings experienced a daily average maximum light intensity of $\approx 800\,\upmu \hbox {mol}\,\hbox {PPFD} \,\hbox {m}^{-2}\,\hbox {s}^{-1}$. Measurements were performed on single mature leaves. For each leaf, gas-exchange and pulse-amplitude modulated (PAM) fluorescence were measured with a LI-6800 system (LI-COR, Inc., Lincoln, NE, USA). This system was used because it permits simultaneous and quantitative analysis of electron transport (via PAM fluorescence) and carbon metabolism (via gas-exchange). The measurement protocol was designed to ensure that photosynthesis could be assayed at steady-state, and across transitions between light-limited and light-saturated regimes. All of the measurements were conducted at 25 $^{\circ }\hbox {C}$ leaf temperature, and 400 ppmv $\hbox {CO}_2$, 55% relative humidity, and 20.9% $\hbox {O}_2$ in the cuvette. Each measurement began from an overnight dark-acclimated state. Over an 8 h period, the light intensity was increased to a peak light intensity of 200, 400, 800, 1600, or $2400\,\upmu \hbox {mol}\,\hbox {PPFD}\, \hbox {m}^{-2}\,\hbox {s}^{-1}$ and then decreased back to darkness in a sine wave pattern (Fig. 2a). The peak exposure intensities were selected to span from below to above the growth light regime, and the rates and directions of change were selected to mimic mean diurnal cycles. The actinic light was provided as mixture of red and blue wavelengths, with blue at 10% up to a cap at $40\,\upmu \hbox {mol}\, \hbox {PPFD}\,\hbox {m}^{-2}\,\hbox {s}^{-1}$ (r90B40). Measurements of PAM fluorescence and gas-exchange were made at 8 min intervals. In the dark, a rectangular flash was used (5000 $\upmu \hbox {mol}\,\hbox {PPFD}\,\hbox {m}^{-2}\,\hbox {s}^{-1}$ for 1 s; Fig. 2b). In the light, a multi-phase flash was used (i.e., three 300 ms phases; first and third phase at 5,000 $\upmu \hbox {mol}\,\hbox {PPFD}\,\hbox {m}^{-2}\,\hbox {s}^{-1}$; second phase ramped down by 25% for determination of $F^{'}_{m}$), followed by 2 s of far-red illumination and a 5 s dark pulse for determination of $F^{'}_{o}$ (Fig. 2c; Markgraf and Berry 1990; Earl and Ennahli 2004; Loriaux et al. 2013; Avenson and Saathoff 2018).

**Fig. 3:**
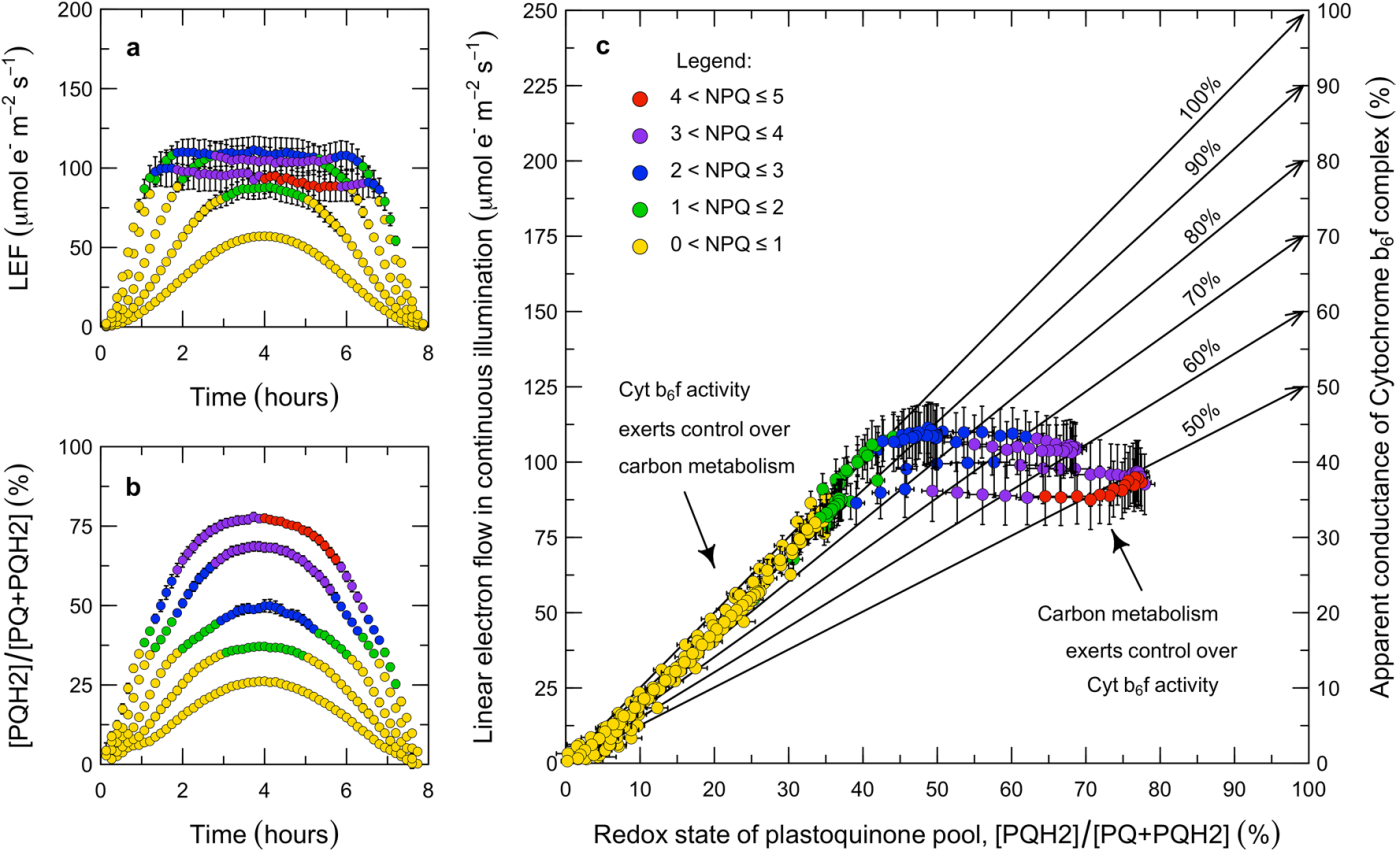
Role of Cytochrome $\hbox {b}_{6}\hbox {f}$ in the control of electron transport during continuous illumination. Under continuous illumination, the relationship between LEF and the redox state of the PQ pool differs between limiting and saturating light intensities (**a**, **b**). Under limiting intensities, LEF is linearly proportional to the redox state of the PQ pool (**a**, **b**) because the apparent conductance of Cyt $\hbox {b}_{6}\hbox {f}$ is maximal (**c**). Once illumination is saturating, LEF is constant and independent of the redox state of the PQ pool (**a**, **b**) because the apparent conductance of Cyt $\hbox {b}_{6}\hbox {f}$ is downregulated (**c**). In these plots, the apparent LEF is the product of the light intensity, *Q*; an estimated absorption cross-section, $\alpha _2 = 0.85 \cdot 0.5$; and the photochemical yield, $\varPhi _{P2}$ (Genty et al. 1989). The apparent redox state of the PQ pool is $1 - qL$ (Kramer et al. 2004). The apparent conductance of Cyt $\hbox {b}_{6}\hbox {f}$ is estimated by extrapolating from the LEF that corresponds to the completely oxidized state of the PQ pool, through a given observation, to the LEF that corresponds to the completely reduced state of the PQ pool (sloped lines in **c**; $k_{Lake} = LEF/(1 - qL)$). The responses are grouped by *NPQ*, given as $F^{}_m/F^{\prime }_m - 1$ (Bilger and Björkman 1990). Each point represents the mean of ${n} = 6$ replicates ± std. error, measured in 8 min increments over an 8 h period (${N} = 1830$)

**Fig. 4:**
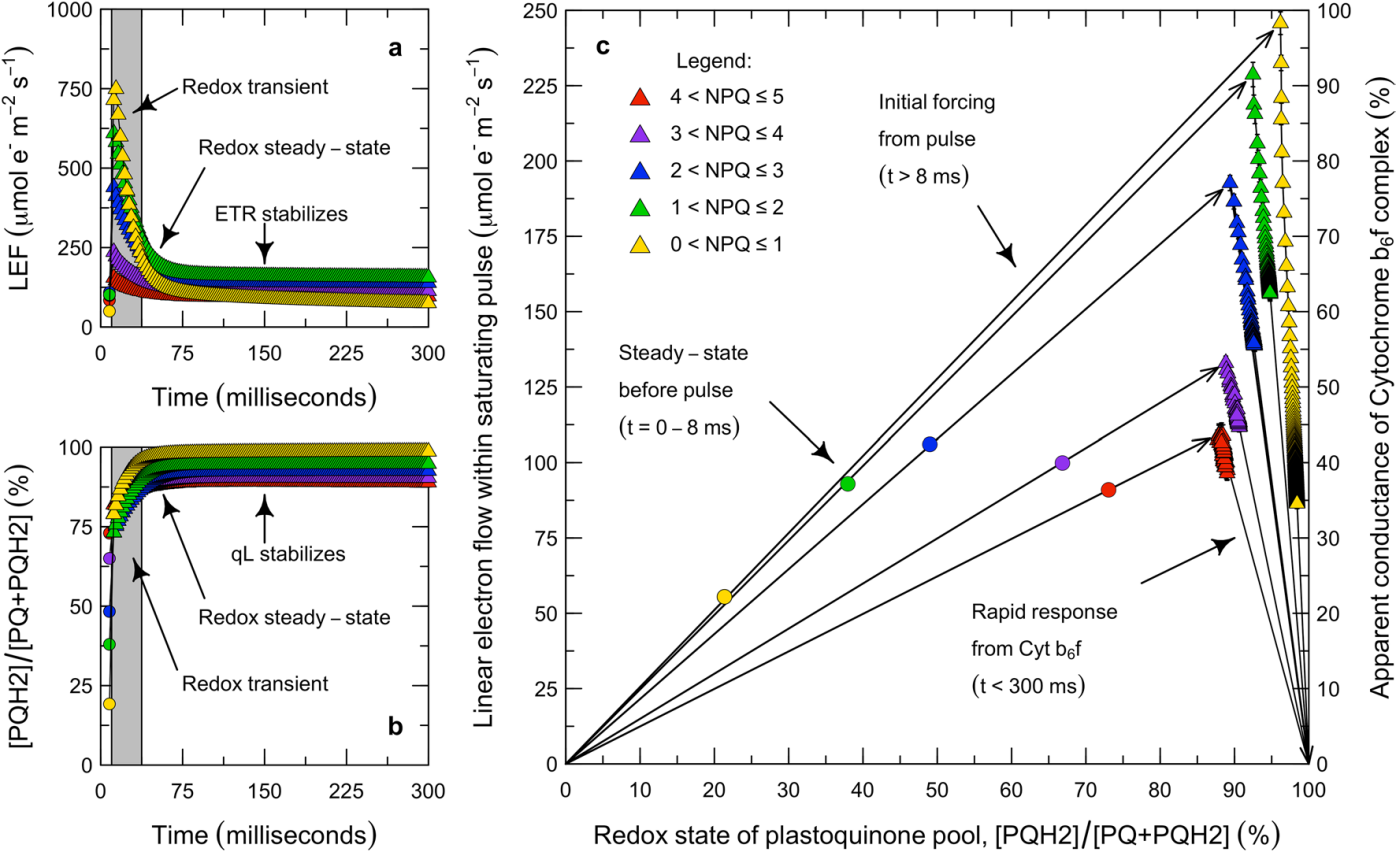
Role of Cytochrome $\hbox {b}_{6}\hbox {f}$ in the control of electron transport during saturating pulses. During each pulse, LEF initially increases (**a**), the PQ pool becomes more reduced (**b**), and then LEF decreases as the apparent conductance of Cyt $\hbox {b}_{6}\hbox {f}$ decreases (**c**). The extent of the surge in LEF, the over-reduction of PQ, and the decrease in Cyt $\hbox {b}_{6}\hbox {f}$ conductance are all inversely proportional to the level of NPQ developed before the pulse (**a**, **b**, **c**). In **c**, the data are filtered to exclude the initial redox transient using the criterion $\varDelta |qL| < 0.0025$ m$\hbox {s}^{-1}$. As in the previous figure, the apparent LEF is the product of the light intensity, *Q*; an estimated absorption cross-section, $\alpha _2 = 0.85 \cdot 0.5$; and the photochemical yield, $\varPhi _{P2}$ (Genty et al. 1989). The apparent redox state of the PQ pool is $1 - qL$ (Kramer et al. 2004). The apparent conductance of Cyt $\hbox {b}_{6}\hbox {f}$ is $k_{Lake} = LEF/(1 - qL)$. The responses are grouped by *NPQ*, given as $F^{}_m/F^{\prime }_m - 1$ (Bilger and Björkman 1990). Each point represents the mean ± std. error across all of the observations in a given NPQ group, but in many cases the uncertainties are so small as to be obscured by the points. There were ${n} = 912$, 209, 208, 274, and 83 observations in each of the NPQ groups, from lowest to highest NPQ. This represented 97% of the ramped pulses in Fig. 2c; the remaining 3% were discarded after filtering with quality-control criteria (*N* = 1686 of 1732)

Using the PAM fluorescence and gas-exchange measurements (Fig. 2d–l), we propose a method for diagnosing the control of linear electron flow (LEF). Our analysis is based on the concepts that the steady-state rate of LEF is kinetically limited by the oxidation of reduced plastoquinone at Cyt $\hbox {b}_{6}\hbox {f}$ and that this reaction has a first-order dependence on $\hbox {PQH}_2$ (Stiehl and Witt 1969). With the flux of absorbed light and PAM fluorescence levels, the rate of LEF can be estimated from the photochemical yield of PS II, $\varPhi _{P2}$ (Genty et al. 1989). Assuming a lake-type model for the PS II antennae, the fractional reduction of the plastoquinone pool can be estimated as $1 - qL$ (Kramer et al. 2004). Although the *qL* index is usually discussed in relation to the closure of PS II reaction centers, the redox poise of the PQ/$\hbox {PQH}_2$ pool couples the acceptor side of PS II to the donor side of Cyt $\hbox {b}_{6}\hbox {f}$. Since there is thought to be minimal diffusion limitation between PS II and Cyt $\hbox {b}_{6}\hbox {f}$ (Laisk et al. 2005a; Tikhonov 2013, 2018), $1 - qL$ should also provide a reasonable steady-state approximation of the state of the donor side of Cyt $\hbox {b}_{6}\hbox {f}$. As a result, LEF can be factorized as the product of the fraction of Cyt $\hbox {b}_{6}\hbox {f}$ sites occupied by reduced plastoquinone (%) and the rate at which each reduced site turns over ($\hbox {mol}\,\hbox {m}^{-2}\,\hbox {s}^{-1}$). With this approach, the apparent conductance of Cyt $\hbox {b}_{6}\hbox {f}$ to LEF can be estimated with $k_{Lake} = LEF/(1 - qL)$ for values of $qL < 1$. The control of LEF can then be interpreted in terms of the balance between the excitation pressure on PS II (i.e., which drives electrons into the plastoquinone pool) and the apparent conductance or resistance of Cyt $\hbox {b}_{6}\hbox {f}$ (i.e., which permits electrons to drain from the plastoquinone pool). Our focus on probing the upstream side of Cyt $\hbox {b}_{6}\hbox {f}$ with fluorescence-based measurements of the PQ redox state differentiates this experiment from earlier ones that have probed the downstream side of Cyt $\hbox {b}_{6}\hbox {f}$ using absorbance-based measurements of the redox states of PC and PS I (Laisk et al. 2005a). We have applied this analysis to the steady-state conditions as well as in the transients associated with each PAM flash in the sine wave experiment (Figs. 3 and 4).

#### Experimental analysis

In the steady-state, the apparent conductance of Cyt $\hbox {b}_{6}\hbox {f}$ to LEF can vary between a fully open state and a variably downregulated state (Fig. 3). During the steady-state measurements, we applied PAM flashes in 8 min intervals and used these to calculate the time course of LEF and the poise of PQ/$\hbox {PQH}_2$ over each 8 h sine wave (Fig. 3a, b). The relationship between these two parameters reveals that the apparent conductance of Cyt $\hbox {b}_{6}\hbox {f}$ to LEF differs systematically between limiting versus saturating light (Fig. 3c). Under limiting light, there is a linear relationship between LEF and the poise of PQ/$\hbox {PQH}_2$ which corresponds to Cyt $\hbox {b}_{6}\hbox {f}$ operating at a constant and maximal conductance (Fig. 3c; points corresponding to 100% apparent conductance of Cyt $\hbox {b}_{6}\hbox {f}$). Our interpretation is that this reflects a regulatory regime in which the total light absorption by PS II and PS I is maximized by chloroplast movements, the absorption cross-sections of PS II and PS I are optimized by state transitions, and photosynthetic control of Cyt $\hbox {b}_{6}\hbox {f}$ is relaxed. Under these conditions, LEF proceeds at the rate permitted by the $\hbox {PQH}_2$ supply and the maximum conductance of Cyt $\hbox {b}_{6}\hbox {f}$. This state appears to be maintained as long as Rubisco is being activated and the photosynthetic carbon reduction (PCR) and photosynthetic carbon oxidation (PCO) cycles are consuming all of the available reductant and ATP. Then, a transition occurs at the light saturation point. Under saturating light, the rate of LEF is constant, the plastoquinone pool continues to become reduced, and the apparent conductance of Cyt $\hbox {b}_{6}\hbox {f}$ progressively decreases (Fig. 3c; points corresponding to $<100\%$ apparent conductance of Cyt $\hbox {b}_{6}\hbox {f}$). Our interpretation is that this reflects a regulatory regime in which chloroplast movements decrease excess light absorption by PS II and PS I, psbS-dependent and zeaxanthin-dependent quenching increase heat dissipation from PS II, and photosynthetic control downregulates the conductance of Cyt $\hbox {b}_{6}\hbox {f}$ to LEF. This state appears to maintain LEF constant and independent of light once Rubisco is fully activated and the PCR and PCO cycles have reached their capacity to consume reductant and ATP. Further insight into how this is achieved can be derived from analysis of the PAM flashes.

Within PAM flashes, the rate of LEF can be driven close to the theoretical upper limit imposed by Cyt $\hbox {b}_{6}\hbox {f}$, but the apparent conductance of Cyt $\hbox {b}_{6}\hbox {f}$ to LEF can also be downregulated very rapidly (Fig. 4). During the PAM flashes, time courses of fluorescence levels were recorded at 2 ms intervals. The multi-phase flash protocol was used to determine the true value of $F^{'}_{m}$ such that the lower, apparent $F^{'}_{m}$ could be interpreted as $F^{}_{s}$. We used these to calculate the time course of LEF and *qL* within each of hundreds of flashes, and then aggregated the responses based on the level of NPQ. Over the course of each PAM flash, the reduction of the plastoquinone pool and the rate of LEF both changed (Fig. 4a, b). By design, the duration of the flashes is short enough that all of the forms of NPQ are effectively constant within the flash. If the conductance of Cyt $\hbox {b}_{6}\hbox {f}$ were also constant within the flash, we would expect the rate of LEF to change only along a line passing through (0, 0) and the point indicating the LEF and PQ/$\hbox {PQH}_2$ poise that prevailed before the flash—but this is not what is observed (Fig. 4c). The overall responses have three phases. In the first phase, the rate of LEF through PS II increases several-fold, out of equilibrium with the rate of LEF through Cyt $\hbox {b}_{6}\hbox {f}$ (Fig. 4a). In this phase, the PQ/$\hbox {PQH}_2$ pool becomes strongly reduced (Fig. 4b). We have excluded this redox transient from Fig. 4c because it cannot be interpreted in terms of the rate of LEF through Cyt $\hbox {b}_{6}\hbox {f}$. In the second phase, redox equilibrium is established between PS II and the PQ/$\hbox {PQH}_2$ pool, and the rate of LEF through PS II and Cyt $\hbox {b}_{6}\hbox {f}$ reaches a value determined by the previous $k_{Lake}$ and the new poise of the PQ/$\hbox {PQH}_2$ pool (i.e., along the upward vectors on the left in Fig. 4c). In the third phase, the rate of LEF through PS II and Cyt $\hbox {b}_{6}\hbox {f}$ decreases rapidly while there are small additional increases in the redox level of the PQ/$\hbox {PQH}_2$ pool (i.e., along the downward vectors on the right in Fig. 4c). By 300 ms into the flash, LEF has decreased to a value only slightly higher than the original value (Fig. 4a)—despite the fact that the PQ pool is much more reduced (Fig. 4b). This indicates that the apparent conductance of Cyt $\hbox {b}_{6}\hbox {f}$ to LEF is now much lower than at the beginning of the pulse. Our interpretation is that this time course of events reveals a regulatory feedback which is closely coupled to the poise of energy carriers in the stroma, and may represent the same mechanism of photosynthetic control of Cyt $\hbox {b}_{6}\hbox {f}$ that is evident in the steady-state analysis.

#### Experimental discussion

These analyses demonstrate a dual role of Cyt $\hbox {b}_{6}\hbox {f}$ in photosynthesis: it presents a passive resistance to LEF when light is limiting (Fig. 3), and it functions as a current-limiting element when light is saturating (Fig. 4). When Cyt $\hbox {b}_{6}\hbox {f}$ is in the minimal resistance (or maximal conductance) state, extrapolation to complete reduction of the plastoquinone pool can be used to estimate the maximum catalytic activity of this enzyme in vivo (Fig. 3c), and flash-induced reduction of the plastoquinone pool can be used to transiently drive the enzyme at this maximum activity (Fig. 4c). It is important to note that this approach is expected to somewhat underestimate ${V}_{\mathrm{max}}$ because the connectivity of the PS II antennae is thought to be less than the pure ‘lake’-type model, and because a small fraction of the total electron flow through Cyt $\hbox {b}_{6}\hbox {f}$ is thought to participate in a cyclic pathway around PS I (CEF1) under limiting light intensities. However, even with this caveat, the ${V}_{\mathrm{max}}$ is on the order of 2$\times$ higher than the maximal rates of LEF observed under continuous illumination. In this respect, there is an important parallel between the expression and regulation of Cyt $\hbox {b}_{6}\hbox {f}$ and Rubisco. It is also possible to estimate the maximum activity of Rubisco in vivo using extrapolation or rapid impulses of $\hbox {CO}_2$, and this demonstrates maximum catalytic activity that is much higher than the maximal rates of $\hbox {CO}_2$ assimilation that are observed under saturating light and a constant and saturating level of $\hbox {CO}_2$ (Laisk and Oya 1975). Just as feedback drives downregulation of Rubisco under conditions where triose phosphate utilization becomes limiting, feedback also drives downregulation of Cyt $\hbox {b}_{6}\hbox {f}$ under conditions where RuBP utilization becomes limiting.

This perspective suggests that the ${V}_{\mathrm{max}}$ values of Cyt $\hbox {b}_{6}\hbox {f}$ and Rubisco represent the primary limits on the activities of electron transport and carbon metabolism, respectively, and that these limits structure the regulatory feedbacks that coordinate fluxes through the photosynthetic system—most notably, photosynthetic control. Traditionally, photosynthetic control has been assumed to act on the kinetic bottleneck at Cyt $\hbox {b}_{6}\hbox {f}$ via a regulatory sequence in which: (i) accumulation of ATP or depletion of inorganic phosphate slows proton efflux through the ATP synthase, (ii) such that the thylakoid lumen becomes acidified and (iii) exerts backpressure on the proton-coupled electron transfer at Cyt $\hbox {b}_{6}\hbox {f}$ (West and Wiskich 1968). However, it has long been a matter of debate whether this mechanism is engaged in vivo during steady-state photosynthesis under normal environmental conditions (i.e., at the transition to saturating light, under ambient $\hbox {CO}_2$ and $\hbox {O}_2$, and at permissive temperatures; Weis et al. 1987; Foyer et al. 1990; Genty and Harbinson 1996; Baker et al. 2007; Foyer et al. 2012; Tikkanen et al. 2012; Johnson et al. 2014; Finazzi et al. 2016). To date, some observations have been interpreted as evidence that feedback regulation of electron transport does not in fact occur under these conditions (e.g., Harbinson and Hedley 1989; Laisk and Oja 1994; Kramer et al. 1999). Others have been interpreted as evidence that feedback regulation occurs under these conditions, but operates through a redox-based mechanism rather than $\varDelta$pH-based mechanism (e.g., Ott et al. 1999; Golding and Johnson 2003; Hald et al. 2008). Still others have been interpreted as evidence that feedback regulation not only occurs under these conditions but also operates through $\varDelta$pH- and/or $\varDelta \psi$-based mechanisms—much as traditionally proposed (e.g., Laisk et al. 2005a; Takizawa et al. 2007; Kanazawa et al. 2017). In this context, our results provide new perspective because they reveal that the onset of photosynthetic control at the light saturation point is abrupt (Fig. 3c) and feedback can induce photosynthetic control extremely rapidly, on the order of milliseconds (Fig. 4c). These features of photosynthetic control cannot be easily reconciled with the conventional pH-driven mechanism, and seem to be more consistent with a redox-based mechanism.

The method of diagnosing photosynthetic control that we have introduced above provides a new basis for assessing how photosynthetic control is achieved in vivo and how it interacts with other forms of feedback regulation—particularly NPQ and CEF1. Since NPQ downregulates PS II and thereby restricts the flow of electrons through the intersystem chain to PS I, it is often interpreted as having a photoprotective function. However, it is difficult to reconcile this view with the observations that a significant fraction of NPQ develops before light saturation (Fig. 2h), that NPQ does not change abruptly at the light saturation point (Fig. 3a), and that NPQ does not prevent the PQ pool from continuing to become reduced above the light saturation point (Fig. 3b, c). In combination, these observations suggest that NPQ functions to control the excitation balance of PS II relative to PS I, rather than the absolute excitation pressure on PS II. In turn, this leads to a new perspective from which to consider CEF1. While flux through CEF1 is generally thought to be a few percent of LEF under limiting light, CEF1 fluxes equivalent to LEF fluxes have been reported under saturating light (e.g., Heber and Walker 1992; Golding and Johnson 2003; Miyake et al. 2005) and it has been proposed that in such large fluxes the connection from Fd to PQ must be mediated directly by Cyt $\hbox {b}_{6}\hbox {f}$ (e.g., Joliot and Joliot 2006; Joliot and Johnson 2011; Nawrocki et al. 2019). In these reports, CEF1 is interpreted as functioning to build up the proton motive force for production of ATP, induction of NPQ, and/or engagement of photosynthetic control of Cyt $\hbox {b}_{6}\hbox {f}$. While the results of the sine wave experiment are potentially consistent with these interpretations, they also point to another possibility: that direct competition from electrons in CEF1 might modulate the conductance of Cyt $\hbox {b}_{6}\hbox {f}$ to LEF, and NPQ might have a critical role in balancing excitation of the photoacts in a way that facilitates CEF1. As it is difficult to differentiate between these possibilities with qualitative approaches alone, we now turn to the development of a quantitative model.

## Model

In ‘Introduction’, we introduced the role and kinetic properties of Cyt $\hbox {b}_{6}\hbox {f}$ in the context of the overall functioning of the photosynthetic system of a leaf (Fig. 1). This also introduced several unresolved questions about how the system is integrated and interrelated. In ‘Model’, we now turn to constructing a model that captures the role of Cyt $\hbox {b}_{6}\hbox {f}$ and the ATP synthase in linking PS I and PS II with their associated pigment systems to the energy consuming reactions of carbon metabolism. The model presentation is organized into five sections, which describe the governing equations for electron transport and carbon metabolism (‘Governing equations’), the rate equations for electron transport (‘Electron transport rate equations’), the overall solution (‘Model solution’), an example of inverse fitting (‘Model inversion’), and key predictions from forward simulations (‘Model simulations’).

### Governing equations

In this section, we develop governing equations which define the steady-state fluxes linking electron transport and carbon metabolism. The governing equations are based on the concept that the production of Fd, NADPH, and ATP must be closely coordinated with the rate at which these compounds can be used in metabolism across a wide range of environmental conditions (Fig. 1; yellow summation point). It follows from this that linear electron flow through PS II, Cyt $\hbox {b}_{6}\hbox {f}$, and PS I (LEF) and cyclic electron flow through PS I and Cyt $\hbox {b}_{6}\hbox {f}$ (CEF1) are regulated in such a way as to provide Fd, NADPH, and ATP at a rate that matches the capacity of the PCR and PCO cycles to serve as sinks for these metabolites. One might model this by describing how the steady-state concentrations of ATP, NADPH, and Fd feedback to regulate the proportions of LEF and CEF1 under a particular condition (e.g., Laisk et al. 2009a; Zhu et al. 2013; Tikhonov and Vershubskii 2014; Morales et al. 2018b; Matuszyńska et al. 2019). However, we have modeled the steady-state fluxes of ATP, NADPH, and Fd by working backwards from the kinetic description of carbon metabolism provided by the Farquhar et al. (1980) model. With this approach, we are able to quantify the minimum required rates of LEF and CEF1 without having complete knowledge of all of the intermediate mechanisms which coordinate electron transport and carbon metabolism.

#### Reaction stoichiometry

In this model, we adopt the same stoichiometries to characterize NADPH, Fd, and ATP supply and demand as introduced by Farquhar et al. (1980). We begin by defining two metabolic pathways that can supply energy: LEF and CEF1. The reaction sequences for LEF to NADPH and Fd are given by:

1a $$\begin{aligned}&4 \hbox {hv}_{\mathrm{P}680 [\mathrm{t}]} + 4 \hbox {hv}_{\mathrm{P}700 [\mathrm{t}]} + 2 {\hbox {H}_2\hbox {O}}_{[\mathrm{l}]} + 2 \hbox {NADP}^+_{[\mathrm{s}]} + \\&\quad \hbox {n} \cdot (\hbox {ADP}_{[\mathrm{s}]} + \hbox {P}_{{\mathrm{i [s]}}} + \hbox {H}^+_{[\mathrm{s}]}) \rightarrow \nonumber \\&\quad {\hbox {O}_2}_{[\mathrm{l}]} + 2 \hbox {NADPH}_{[\mathrm{s}]} + 2 \hbox {H}^+_{[\mathrm{s}]} + \hbox {n} \cdot (\hbox {ATP}_{[\mathrm{s}]} + {\hbox {H}_2\hbox {O}}_{[\mathrm{s}]}) \nonumber \end{aligned}$$

1b $$\begin{aligned}&4 \hbox {hv}_{\mathrm{P}680 [\mathrm{t}]} + 4 \hbox {hv}_{\mathrm{P}700 [\mathrm{t}]} + 2 {\hbox {H}_2\hbox {O}}_{[\mathrm{l}]} + 4 {\hbox {Fd}_{\mathrm{ox}}}_{[\mathrm{s}]} + \\&\quad \hbox {n} \cdot (\hbox {ADP}_{[\mathrm{s}]} + \hbox {P}_{{\mathrm{i [s]}}} + \hbox {H}^+_{\mathrm{[s]}}) \rightarrow \nonumber \\&\quad {\hbox {O}_2}_{[\mathrm{l}]} + 4 {\hbox {Fd}_{\mathrm{red}}}_{[\mathrm{s}]} + 4 \hbox {H}^+_{[\mathrm{s}]} + \hbox {n} \cdot (\hbox {ATP}_{[\mathrm{s}]} + {\hbox {H}_2\hbox {O}}_{[\mathrm{s}]}) \nonumber \end{aligned}$$

respectively. The reaction sequence for CEF1 is given by:

2 $$\begin{aligned}&8 \hbox {hv}_{{\mathrm{P700 [t]}}} + \hbox {n} \cdot (\hbox {ADP}_{[\mathrm{s}]} + \hbox {P}_{{\mathrm{i [s]}}} + \hbox {H}^+_{[\mathrm{s}]}) \rightarrow \\&\quad \hbox {n} \cdot (\hbox {ATP}_{[\mathrm{s}]} + \hbox {H}_2\hbox {O}_{[\mathrm{s}]}) \nonumber \end{aligned}$$

respectively. In Eqs. 1 and 2, the subscripts [t], [l], and [s] indicate localization to the thylakoid membrane, thylakoid lumen, and chloroplast stroma, and the value of *n* depends on the assumptions about proton production and consumption.

We consider three metabolic pathways that can consume energy: photosynthetic carbon reduction (PCR) cycle, photosynthetic carbon oxidation (PCO) cycle, and reduced carbon export. The net reaction for the PCR reaction sequence is given by:

3 $$\begin{aligned}&3 {\hbox {CO}_2}_{[\mathrm{s}]} + 6 \hbox {NADPH}_{[\mathrm{s}]} + 9 \hbox {ATP}_{[\mathrm{s}]} + 5 {\hbox {H}_2\hbox {O}}_{[\mathrm{s}]} \rightarrow \\&\quad \hbox {GAP}_{[\mathrm{s}]} + 6 \hbox {NADP}^{+}_{[\mathrm{s}]} + 9 \hbox {ADP}_{[\mathrm{s}]} + 8 \hbox {P}_{{\mathrm{i [s]}}} + 3 \hbox {H}^{+}_{\mathrm{[s]}} \nonumber \end{aligned}$$

where the coefficients correspond to three mol Rubisco carboxylase reactions and GAP is D-glyceraldehyde-3-phosphate. Accounting for partial regeneration of RuBP, the net reaction for the PCO reaction sequence is given by:

4 $$\begin{aligned}&\hbox {GAP}_{{\mathrm{[s]}}} + 21 \hbox {ATP}_{\mathrm{[s]}} + 9 \hbox {NADPH}_{\mathrm{[s]}} + 6 {\hbox {Fd}_{\mathrm{red}}}_{\mathrm{[s]}} +\\&\quad 10 {\hbox {H}_2\hbox {O}}_{{\mathrm{[s]}}} + 3 {\hbox {H}_2\hbox {O}}_{{\mathrm{[m]}}} + 6 {\hbox {O}_2}_{{\mathrm{[s]}}} + 3 {\hbox {O}_2}_{\mathrm{[p]}} \rightarrow \nonumber \\&\quad 21 \hbox {ADP}_{{\mathrm{[s]}}} + 9 \hbox {NADP}^{+}_{{\mathrm{[s]}}} + 6 {\hbox {Fd}_{{\mathrm{ox}}}}_{\mathrm{[s]}} + 22 \hbox {P}_{\mathrm{i [s]}} + \nonumber \\&\quad 6 \hbox {H}^{+}_{{\mathrm{[s]}}} + 6 {\hbox {H}_2\hbox {O}}_{{\mathrm{[p]}}} + 3 {\hbox {CO}_2}_{\mathrm{[m]}} \nonumber \end{aligned}$$

where the coefficients correspond to six mol Rubisco oxygenase reactions, and the subscripts [m] and [p] indicate localization to the mitochondrion and peroxisome. Under conditions where triose phosphate production in the PCR cycle exceeds triose phosphate consumption in the PCO cycle, triose phosphate is exported to storage/transport compounds. If export is to the cytosol for sucrose synthesis, the net reaction is given by:

5 $$\begin{aligned}&4 \hbox {GAP}_{{\mathrm{[s]}}} + 4 {\hbox {H}_2\hbox {O}}_{\mathrm{[c]}} + \hbox {ATP}_{{\mathrm{[c]}}} \rightarrow \\&\quad 4 \hbox {P}_{{\mathrm{i [s]}}} + 1 \hbox {P}_{{\mathrm{i [c]}}} + \hbox {ADP}_{{\mathrm{[c]}}} + \hbox {H}^+_{{\mathrm{[c]}}} + 1 \hbox {SUCR}_{{\mathrm{[c]}}} \nonumber \end{aligned}$$

where the coefficients correspond to four mol GAP consumption, the subscript [c] indicates localization to the cytosol, and SUCR represents sucrose. Since the ATP-requiring step in sucrose synthesis occurs in the cytosol, we assume that this ATP is supplied by mitochondrial electron transport rather than chloroplast electron transport.

#### Energy, mass, and charge balance

Traditionally, the stoichiometries described in the previous section have been applied in models with the assumption that the photosynthetic system operates at one ‘pin’ of the energy balance, i.e., either in a reductant-limited or an ATP-limited state (von Caemmerer 2000). While this is a reasonable assumption during transient adjustments to altered conditions, it is not satisfactory for characterization of the steady-state. Here, we develop an alternate approach based on the concept that energy supply and demand are dynamically coordinated by regulatory interactions that continuously correct transient imbalances in the production and consumption of Fd, NADPH, and ATP, such that in the steady-state the Fd, NADPH, and ATP balances are satisfied simultaneously.

From Eqs. 1 and 2, the rates of Fd, NADPH, and ATP export from the electron transport system to carbon metabolism are given by:

6a $$\begin{aligned}&J_{Fd} + J_{NADPH} \cdot 2 = J_{P680} \end{aligned}$$

6b $$\begin{aligned}&J_{ATP} = J_{P680} \cdot n_L + ( J_{P700} - J_{P680}) \cdot n_C \end{aligned}$$

where $J_{P680}$ is the total rate of LEF through PS II ($\hbox {mol} \, \hbox {e}^{-} \,\hbox {m}^{-2}\,\hbox {s}^{-1}$), $J_{P700}$ is the total rate of LEF and CEF1 through PS I ($\hbox {mol}\, \hbox {e}^{-} \,\hbox {m}^{-2}\,\hbox {s}^{-1}$), $J_{Fd}$ and $J_{NADPH}$ are the rates of $\hbox {Fd}$ and ${\hbox {NADPH}}$ export (mol Fd or NADPH $\hbox {m}^{-2}\,\hbox {s}^{-1}$), $J_{ATP}$ is the rate of ATP export (mol ATP $\hbox {m}^{-2}\,\hbox {s}^{-1}$) and $n_L$ and $n_C$ are composite coupling efficiencies for LEF and CEF1 (mol ATP produced $\hbox {mol}^{-1}$ electrons). N.B., the coupling efficiencies account for the stoichiometry linking electron flow to proton pumping into the lumen, as well as the stoichiometry linking proton efflux via the ATP synthase to ATP synthesis. From Eqs. 3 and 4, the rates of Fd, NADPH, and ATP consumption by carbon metabolism are given by:

7a $$\begin{aligned} J_{NADPH}&= 2 \cdot V_c + 1.5 \cdot V_o \end{aligned}$$

7b $$\begin{aligned} J_{Fd}&= V_o \end{aligned}$$

7c $$\begin{aligned} J_{ATP}&= 3 \cdot V_c + 3.5 \cdot V_o \end{aligned}$$

where $V_c$ and $V_o$ are the carboxylation and oxygenation rates of Rubisco ($\hbox {mol}\,\hbox {CO}_2$ or $\hbox {O}_2\,\hbox {m}^{-2}\, \hbox {s}^{-1}$).

Combining Eqs. 6 and 7, it follows that:

8a $$\begin{aligned} J_{P680}&= 4 \cdot V_c + 4 \cdot V_o \end{aligned}$$

8b $$\begin{aligned} J_{P700}&= (3 \cdot V_c + 3.5 \cdot V_o - J_{P680} \cdot n_L)/n_C + J_{P680} \end{aligned}$$

when the Fd, NADPH, and ATP budgets are balanced simultaneously. Under this condition, the overall reaction for photosynthesis is given by:

9 $$\begin{aligned}&\hbox {n hv}_{{\mathrm{[t]}}} + 12 {\hbox {CO}_2}_{{\mathrm{[s]}}} + 11 {\hbox {H}_2\hbox {O}}_{{\mathrm{[s, c]}}} \rightarrow \\&\quad 12 {\hbox {O}_2}_{\mathrm{[s]}} + 1 \hbox {SUCR}_{\mathrm{[c]}} \nonumber \end{aligned}$$

which results from combining Eqs. 1–5. In this expression, the value of *n* varies with the ratio of PCO to PCR cycle activity, but there is always a 1:1 $\hbox {CO}_2$:$\hbox {O}_2$ exchange ratio.

#### Relating electron transport to gas-exchange

The rates of PS II and PS I electron transport in Eq. 8 can be linked directly to the rate of $\hbox {CO}_2$ assimilation in Eq. 9 through the gas-exchange expressions of Farquhar et al. (1980). The observed net rate of $\hbox {CO}_2$ assimilation, *A*, is given by:

10 $$\begin{aligned} A = A_g - R_d \end{aligned}$$

where $A_g$ is the gross rate of $\hbox {CO}_2$ assimilation and $R_d$ is the rate of day respiration, i.e., mitochondrial $\hbox {CO}_2$ release other than that associated with photorespiration (all in $\hbox {mol}\,\hbox {CO}_2\,\hbox {m}^{-2}\, \hbox {s}^{-1}$). The value of $A_g$ is given by:

11 $$\begin{aligned} \quad A_g = V_c - 0.5 \cdot V_o \end{aligned}$$

where $V_c$ and $V_o$ are the carboxylation and oxygenation rates of Rubisco ($\hbox {mol}\,\hbox {CO}_2$ or $\hbox {O}_2\,\hbox {m}^{-2}\, \hbox {s}^{-1}$), and 0.5 is the ratio between $\hbox {CO}_2$ release and $\hbox {O}_2$ uptake in photorespiration (mol $\hbox {CO}_2\, \hbox {mol}^{-1}\,\hbox {O}_2$). Equation 11 can be linked directly to electron transport by defining:

12 $$\begin{aligned} \dfrac{V_o}{V_c} = \dfrac{1}{S} \cdot \dfrac{O}{C} \end{aligned}$$

where *S* is the specificity of Rubisco for carboxylation relative to oxygenation ($\hbox {mol}\,\hbox {CO}_2\,\hbox {mol}^{-1}\,\hbox {O}_2$), and *O* and *C* are the partial pressures of $\hbox {O}_2$ and $\hbox {CO}_2$ in the chloroplast (bar). In theory, the value of *S* is given by:

13 $$\begin{aligned} S = \dfrac{k_c}{K_c} \cdot \dfrac{K_o}{k_o} \end{aligned}$$

where $k_c$ and $k_o$ are the catalytic constants of Rubisco for $\hbox {CO}_2$ and $\hbox {O}_2$ ($\hbox {mol}\,\hbox {CO}_2$ or $\hbox {O}_2$ $\hbox {mol}^{-1}$ sites $\hbox {s}^{-1}$), and $K_c$ and $K_o$ are the Michaelis-Menten constants for $\hbox {CO}_2$ and $\hbox {O}_2$ (bar). However, *S* can be measured directly without evaluating all of the individual constants. The value of *S* determines the $\hbox {CO}_2$ compensation point, $\varGamma_{*}$, which is given by:

14 $$\begin{aligned} \varGamma _{*} = \dfrac{1}{2} \cdot \dfrac{O}{S} \end{aligned}$$

and is defined as the chloroplast $\hbox {pCO}_2$ at which uptake of $\hbox {CO}_2$ via carboxylase activity is balanced with release of $\hbox {CO}_2$ from oxygenase activity. Combining Eq. 8 with Eqs. 10–14 then yields:

15a $$\begin{aligned}&J_{P680} = (A + R_d) \cdot \left( \dfrac{4 + 8 \cdot \varGamma _{*}/C}{1 - \varGamma _{*}/C}\right) \end{aligned}$$

15b $$\begin{aligned}&J_{P700} = J_{P680} \cdot \eta \end{aligned}$$

15c $$\begin{aligned}&\nonumber \\&\eta = 1 - \dfrac{n_L}{n_C} + \dfrac{3 + 7 \cdot \varGamma _{*}/C}{(4 + 8 \cdot \varGamma _{*}/C) \cdot n_C} \end{aligned}$$

where the rates of PS II and PS I electron transport are related to *A* because LEF and CEF1 are coordinated with the activity of the PCR and PCO cycles. Note that Eq. 15c represents a general form of an expression presented by Farquhar and von Caemmerer (1981), i.e., where the difference is that the $n_L$ and $n_C$ parameters allow for the continuing uncertainties regarding coupling between electron flow and ATP production. From here, the next step is to develop expressions which relate electron transport to PAM fluorescence.

### Electron transport rate equations

In this section, we develop rate equations describing the kinetics of electron transport through Cyt $\hbox {b}_{6}\hbox {f}$, PS I, and PS II. The approach we present is novel but is inspired by that of Loriaux et al. (2013) and Rubin and Riznichenko (2014), and interested readers are advised to consult these references for detailed background. As the latter authors discuss, rate equations for electron transport should provide the simplest description of the functional states of the relevant complexes that can capture the major kinetic characteristics of the target flux in a realistic way—but the correct formulation is inherently tied to the spatial and temporal scale of analysis. The rate equations in this section are designed for description of steady-state photosynthesis at the leaf scale. They are based on the concept that at this scale the dynamics of electron transport are limited by two regulated properties: the distribution of excitation between PS I and PS II and the maximum activity of Cyt $\hbox {b}_{6}\hbox {f}$ (Fig. 1; blue photocells and transistor symbol). For PS II and PS I, we describe the reaction centers as cycling between ‘open’ and ‘closed’ states. This two-state abstraction is derived from the fact that during steady-state electron transport the vast majority of PS II reaction centers equilibrate with neutral donor and acceptor components (‘open’) or neutral donor and reduced acceptor components (‘closed’), while most PS I reaction centers equilibrate with neutral donor and acceptor components (‘open’) or oxidized donor and neutral acceptor components (‘closed’). Since the reduction of the acceptor component of PS II and the oxidation of the donor component of PS I are both consequences of the kinetic bottleneck at Cyt $\hbox {b}_{6}\hbox {f}$, we begin by defining the rate equations for Cyt $\hbox {b}_{6}\hbox {f}$.

#### Cytochrome $\hbox {b}_{6}\hbox {f}$

In LEF and CEF1, Cyt $\hbox {b}_{6}\hbox {f}$ mediates the transfer of electrons from $\hbox {PQH}_2$ to ${\hbox {PC}_{{\mathrm{ox}}}}$, and couples this electron transfer to proton pumping from the stroma into the lumen. We model the turnover of Cyt $\hbox {b}_{6}\hbox {f}$ in terms of the $\hbox {PQH}_2$ occupancy of the $\hbox {Q}_{\mathrm{p}}$ site and the rate at which electrons can pass from $\hbox {PQH}_2$, through the Rieske iron-sulfur cluster and Cyt f, to ${\hbox {PC}_{\mathrm{ox}}}$. This step is considered to be the primary kinetic bottleneck in both LEF and CEF1. The total concentration of Cyt $\hbox {b}_{6}\hbox {f}$ is denoted $D^{}_{CB6F}$, and the concentration of Cyt $\hbox {b}_{6}\hbox {f}$ with the $\hbox {Q}_{\mathrm{p}}$ site occupied by $\hbox {PQH}_2$ is denoted $D^{*}_{CB6F}$ (mol $\hbox {m}^{-2}$). We represent the rate of plastoquinol oxidation with a first-order rate constant that describes the rate at which electrons can pass from $\hbox {PQH}_2$ to ${\hbox {PC}_{{\mathrm{ox}}}}$. This rate constant is denoted $k^{*}_{CB6F}$, and has a maximum value that is denoted $k_q$ ($\hbox {mol}\,\hbox {e}^{-}\,\hbox {mol}^{-1}$ sites $\hbox {s}^{-1}$). With this terminology, the rate of electron transport through Cyt $\hbox {b}_{6}\hbox {f}$ is given by:

16 $$\begin{aligned} J_{CB6F} = D^{*}_{CB6F} \cdot k^{*}_{CB6F} \end{aligned}$$

where $J_{CB6F}$ is the electron transport rate through Cyt $\hbox {b}_{6}\hbox {f}$ (mol $\hbox {e}^{-}$ $\hbox {m}^{-2}$ $\hbox {s}^{-1}$), $D^{*}_{CB6F}$ is the concentration of Cyt $\hbox {b}_{6}\hbox {f}$ with the $\hbox {Q}_{\mathrm{p}}$ site occupied by $\hbox {PQH}_2$ (mol $\hbox {m}^{-2}$), and $k^{*}_{CB6F}$ is the turnover constant for those sites ($\hbox {mol}\,\hbox {e}^{-}\, \hbox {mol}^{-1}$ sites $\hbox {s}^{-1}$). Equation 16 leads to a definition of the lower and upper limits on potential electron transport: the lower limit is reached when all of the Cyt $\hbox {b}_{6}\hbox {f}$ sites are occupied by PQ and ${\hbox {PC}_{{\mathrm{red}}}}$, whereas the upper limit is reached when all of the Cyt $\hbox {b}_{6}\hbox {f}$ sites are occupied by $\hbox {PQH}_2$ and ${\hbox {PC}_{{\mathrm{ox}}}}$ ($D^{*}_{CB6F} \rightarrow D^{}_{CB6F}$) and each site turns over at the maximum rate ($k^{*}_{CB6F} \rightarrow k_q$). While the lower limit is simply zero, the upper limit is given by:

17 $$\begin{aligned} V_{max\ (CB6F)} = D^{}_{CB6F} \cdot k_q \end{aligned}$$

where $V_{max\ (CB6F)}$ is the maximum activity of Cyt $\hbox {b}_{6}\hbox {f}$ ($\hbox {mol}\,\hbox {e}^{-}\,\hbox {m}^{-2}$ $\hbox {s}^{-1}$). Under in vitro conditions, the maximum activity of Cyt $\hbox {b}_{6}\hbox {f}$ can be measured using assays with purified Cyt $\hbox {b}_{6}\hbox {f}$ and electron donor/acceptor pairs. Under in vivo conditions, the maximum activity of Cyt $\hbox {b}_{6}\hbox {f}$ can also be estimated from PAM fluorescence measurements at limiting light intensities using extrapolation to complete reduction of plastoquinone and flash-induced reduction of plastoquinone (e.g., see Figs. 3 and 4). Since Cyt $\hbox {b}_{6}\hbox {f}$ turnover kinetically restricts the rates of electron withdrawal from the PQ/$\hbox {PQH}_2$ pool and donation to the ${\hbox {PC}_{{\mathrm{ox}}}}$/${\hbox {PC}_{{\mathrm{red}}}}$ pool, Eqs. 16 and 17 provide the foundation for describing electron transport through PS I and PS II.

#### Photosystem I

In LEF and CEF1, PS I receives electrons from Cyt $\hbox {b}_{6}\hbox {f}$ via ${\hbox {PC}_{{\mathrm{red}}}}$, and donates electrons to ${\hbox {Fd}_{{\mathrm{ox}}}}$. In the model, each PS I photosynthetic unit includes a donor component, an acceptor component, and an associated antennae complex. The donor component represents the reaction center chlorophyll (P700). The acceptor component represents the special chlorophyll a ($\hbox {A}_{0}$), phylloquinone ($\hbox {A}_{1}$), and iron-sulfur centers ($\hbox {F}_{\mathrm{X}}$-$\hbox {F}_{\mathrm{A}}$-$\hbox {F}_{\mathrm{B}}$). The total concentration of PS I reaction centers is denoted $D^{}_{P700}$, and the concentrations of the open and closed states are denoted $D_{P700}^0$ and $D_{P700}^+$ ($\hbox {mol}\,\hbox {m}^{-2}$). The open state at PS I corresponds to an electron donor/acceptor pair where both components are uncharged (such that excitation has the potential to drive charge separation and electron transfer), whereas the closed state at PS I corresponds to an oxidized electron donor and uncharged electron acceptor pair (which cannot undergo charge separation and electron transfer). By definition, photochemistry occurs only at open PS I reaction centers and constitutive heat loss only occurs at closed PS I reaction centers, such that:

18a $$\begin{aligned} \varSigma K_{P700}^0&= K_{P1} + K_{D1} + K_{F1} \end{aligned}$$

18b $$\begin{aligned} \varSigma K_{P700}^+&= K_{X1} + K_{D1} + K_{F1} \end{aligned}$$

where $\varSigma K_{P700}^0$ is the sum of the rate constants for open PS I reaction centers, $\varSigma K_{P700}^+$ is the sum of the rate constants for closed PS I reaction centers, and the rate constants for photochemistry, constitutive heat loss from closed reaction centers, constitutive heat loss from the antennae, and fluorescence are $K_{P1}$, $K_{X1}$, $K_{D1}$, and $K_{F1}$ ($\hbox {s}^{-1}$). The intrinsic yield of an open or closed center is given by the ratio between a particular rate constant and the sum of the rate constants for all of the possible de-excitation pathways. The overall PS I yields are linked to the state distributions and the intrinsic yields of each state by:

19a $$\begin{aligned} \varPhi _{P1}&= \dfrac{D_{P700}^0}{D^{}_{P700}} \cdot \left( \dfrac{K_{P1}}{\varSigma K_{P700}^0}\right) + \dfrac{D_{P700}^+}{D^{}_{P700}} \cdot \left( \dfrac{0}{\varSigma K_{P700}^+}\right) \end{aligned}$$

19b $$\begin{aligned} \varPhi _{X1}&= \dfrac{D_{P700}^0}{D^{}_{P700}} \cdot \left( \dfrac{0}{\varSigma K_{P700}^0}\right) + \dfrac{D_{P700}^+}{D^{}_{P700}} \cdot \left( \dfrac{K_{X1}}{\varSigma K_{P700}^+}\right) \end{aligned}$$

19c $$\begin{aligned} \varPhi _{D1}&= \dfrac{D_{P700}^0}{D^{}_{P700}} \cdot \left( \dfrac{K_{D1}}{\varSigma K_{P700}^0}\right) + \dfrac{D_{P700}^+}{D^{}_{P700}} \cdot \left( \dfrac{K_{D1}}{\varSigma K_{P700}^+}\right) \end{aligned}$$

19d $$\begin{aligned} \varPhi _{F1}&= \dfrac{D_{P700}^0}{D^{}_{P700}} \cdot \left( \dfrac{K_{F1}}{\varSigma K_{P700}^0}\right) + \dfrac{D_{P700}^+}{D^{}_{P700}} \cdot \left( \dfrac{K_{F1}}{\varSigma K_{P700}^+}\right) \end{aligned}$$

where $\varPhi _{P1}$, $\varPhi _{X1}$, $\varPhi _{D1}$, and $\varPhi _{F1}$ are the overall yields of the whole bed of PS I units for photochemistry, constitutive heat loss from closed reaction centers, constitutive heat loss from the antennae, and fluorescence (mol energy dissipated $\hbox {mol}^{-1}$ energy absorbed).

Rate equations for steady-state electron transport through PS I can now be defined by combining Eqs. 19a and 16. For PS I, the rate of electron transport depends on the balance between light absorption (which controls closure of open reaction centers), and Cyt $\hbox {b}_{6}\hbox {f}$ activity (which controls re-opening of closed reaction centers). When an open PS I reaction center receives excitation, donates electrons to bound $\hbox {Fd}_{{\mathrm{ox}}}$, and the resulting $\hbox {Fd}_{{\mathrm{red}}}$ dissociates, it transitions to a closed state ($P700^0 \rightarrow P700^+$). The rate at which this occurs can be expressed as:

20a $$\begin{aligned} J_{P700}&= D_{P700}^0 \cdot k_{P700}^0 \end{aligned}$$

20b $$\begin{aligned} k_{P700}^0&= \dfrac{Q \cdot \alpha _1}{D^{}_{P700}} \cdot \left( \dfrac{K_{P1}}{\varSigma K_{P700}^0}\right) \end{aligned}$$

where $J_{P700}$ is the rate of PS I electron transport ($\hbox {mol}\,\hbox {m}^{-2}\,\hbox {e}^{-}\,\hbox {s}^{-1}$), $D_{P700}^0$ is the concentration of open PS I centers (mol $\hbox {m}^{-2}$), $k_{P700}^0$ is a first-order turnover constant for open PS I centers ($\hbox {s}^{-1}$), *Q* is the photosynthetically active radiation incident on the leaf (mol incident PPFD $\hbox {m}^{-2}\,\hbox {s}^{-1}$), and $\alpha _{1}$ is the absorbance cross-section associated with the PS I bed (mol PPFD absorbed by PS I $\hbox {mol}^{-1}$ incident PPFD). Analogously, closed PS I centers re-open by accepting electrons from $\hbox {PC}_{{\mathrm{red}}}$ ($P700^+ \rightarrow P700^0$). The rate at which this occurs is linked to Cyt $\hbox {b}_{6}\hbox {f}$ activity:

21a $$\begin{aligned} J_{P700}&= D_{P700}^+ \cdot k_{P700}^+ \end{aligned}$$

21b $$\begin{aligned} k_{P700}^+&= \dfrac{D^{*}_{CB6F} \cdot k_{CB6F}^*}{D_{P700}^+} \end{aligned}$$

where $D_{P700}^+$ is the concentration of closed PS I centers ($\hbox {mol}\, \hbox {m}^{-2}$), $k_{P700}^+$ is a first-order turnover constant for closed PS I centers ($\hbox {s}^{-1}$) and the other terms are as defined above. Accordingly, the rate of electron transport through PS I at any given flux of absorbed light depends on the rate of supply of ${\hbox {PC}_{{\mathrm{red}}}}$ from Cyt $\hbox {b}_{6}\hbox {f}$. In turn, the activity of Cyt $\hbox {b}_{6}\hbox {f}$ depends on the rate of supply of $\hbox {PQH}_2$, derived either via CEF1 from PS I or via LEF from PS II.

#### Photosystem II

PS II initiates LEF by splitting water to release molecular oxygen, protons, and electrons. The protons are released into the lumen, and the electrons are donated to Cyt $\hbox {b}_{6}\hbox {f}$ via PQ. As with PS I, each PS II photosynthetic unit includes a donor component, an acceptor component, and an associated antennae complex. The donor component represents the reaction center chlorophyll (P680). The acceptor component represents pheophytin (Pheo), the primary quinone acceptor ($\hbox {Q}_{\mathrm{A}}$), and the secondary quinone acceptor ($\hbox {Q}_{\mathrm{B}}$). The total concentration of PS II reaction centers is denoted $D^{}_{P680}$, and the concentrations of the open and closed states are denoted $D_{P680}^0$ and $D_{P680}^-$ ($\hbox {mol}\,\hbox {m}^{-2}$). The open state at PS II corresponds to an electron donor/acceptor pair where both components are uncharged (such that excitation has the potential to drive charge separation and electron transfer), whereas the closed state at PS II corresponds to an uncharged electron donor and reduced electron acceptor pair (which cannot undergo charge separation and electron transfer). The fates of excitation at open and closed PS II reaction centers are given by:

22a $$\begin{aligned} \varSigma K_{P680}^0&= K_{P2} + K_{N2} + K_{D2} + K_{F2} + K_{U2} \end{aligned}$$

22b $$\begin{aligned} \varSigma K_{P680}^-&= K_{N2} + K_{D2} + K_{F2} + K_{U2} \end{aligned}$$

where $\varSigma K_{P680}^0$ is the sum of the rate constants for open PS II reaction centers, $\varSigma K_{P680}^-$ is the sum of the rate constants for closed PS II reaction centers, and the rate constants for photochemistry, regulated heat loss in the antennae, constitutive heat loss in the antennae, fluorescence, and inter-unit exciton sharing are $K_{P2}$, $K_{N2}$, $K_{D2}$, $K_{F2}$, and $K_{U2}$ ($\hbox {s}^{-1}$). Two notes are needed about these definitions. First, the $K_{N2}$ parameter is a variable that represents the forms of NPQ that dissipate excess excitation from the PS II antennae as heat, i.e., psbS-dependent (qE) and zeaxanthin-dependent (qZ) quenching. The forms of NPQ like state transitions (qT) and chloroplast movements (qM) are not included in $K_{N2}$, and are instead represented by variation in the $\alpha _1$ and $\alpha _2$ parameters. Second, to describe the effects of exciton migration between photosynthetic units within the PS II bed, it is necessary to define the internal yields of PS II units. The internal yields of PS II units describe the fates of excitation in terms of the fluxes of energy that pass out of the photosynthetic units:

23a $$\begin{aligned} \phi _{P2}&= \dfrac{D_{P680}^0}{D^{}_{P680}} \cdot \left( \dfrac{K_{P2}}{\varSigma K_{P680}^0}\right) + \dfrac{D_{P680}^-}{D^{}_{P680}} \cdot \left( \dfrac{0}{\varSigma K_{P680}^-}\right) \end{aligned}$$

23b $$\begin{aligned} \phi _{N2}&= \dfrac{D_{P680}^0}{D^{}_{P680}} \cdot \left( \dfrac{K_{N2}}{\varSigma K_{P680}^0}\right) + \dfrac{D_{P680}^-}{D^{}_{P680}} \cdot \left( \dfrac{K_{N2}}{\varSigma K_{P680}^-}\right) \end{aligned}$$

23c $$\begin{aligned} \phi _{D2}&= \dfrac{D_{P680}^0}{D^{}_{P680}} \cdot \left( \dfrac{K_{D2}}{\varSigma K_{P680}^0}\right) + \dfrac{D_{P680}^-}{D^{}_{P680}} \cdot \left( \dfrac{K_{D2}}{\varSigma K_{P680}^-}\right) \end{aligned}$$

23d $$\begin{aligned} \phi _{F2}&= \dfrac{D_{P680}^0}{D^{}_{P680}} \cdot \left( \dfrac{K_{F2}}{\varSigma K_{P680}^0}\right) + \dfrac{D_{P680}^-}{D^{}_{P680}} \cdot \left( \dfrac{K_{F2}}{\varSigma K_{P680}^-}\right) \end{aligned}$$

23e $$\begin{aligned} \phi _{U2}&= \dfrac{D_{P680}^0}{D^{}_{P680}} \cdot \left( \dfrac{K_{U2}}{\varSigma K_{P680}^0}\right) + \dfrac{D_{P680}^-}{D^{}_{P680}} \cdot \left( \dfrac{K_{U2}}{\varSigma K_{P680}^-}\right) \end{aligned}$$

where $\phi _{P2}$, $\phi _{N2}$, $\phi _{D2}$, $\phi _{F2}$, and $\phi _{U2}$ are the internal yields of the whole bed of PS II units for photochemistry, regulated heat loss in the antennae, constitutive heat loss in the antennae, fluorescence, and inter-unit exciton sharing (mol energy dissipated $\hbox {mol}^{-1}$ energy absorbed). The internal yields sum to unity because excitons that are lost from one unit are gained by another unit within the PS II bed. Assuming that excitation sharing occurs via a random walk, it can be described by:

24 $$\begin{aligned} \sum _{n=0}^{\infty } \phi _{U2}^n = 1 + \phi _{U2} + \phi _{U2}^2 + \cdots = \dfrac{1}{1-\phi _{U2}} \end{aligned}$$

which is an infinite geometric series. Here, the summation is used to indicate that excitation diffuses through the pigment bed from one photosynthetic unit to the next until it is quenched photochemically, quenched non-photochemically, or released as fluorescence. The overall yields are then defined in terms of the fluxes of energy that pass out of the pigment bed:

25a $$\begin{aligned} \varPhi _{P2}&= \phi _{P2} \cdot (1 + \phi _{U2} + \phi _{U2}^2+ \cdots) = \dfrac{\phi _{P2}}{1-\phi _{U2}} \end{aligned}$$

25b $$\begin{aligned} \varPhi _{N2}&= \phi _{N2} \cdot (1 + \phi _{U2} + \phi _{U2}^2+ \cdots) = \dfrac{\phi _{N2}}{1-\phi _{U2}}\end{aligned}$$

25c $$\begin{aligned} \varPhi _{D2}&= \phi _{D2} \cdot (1 + \phi _{U2} + \phi _{U2}^2+ \cdots) = \dfrac{\phi _{D2}}{1-\phi _{U2}}\end{aligned}$$

25d $$\begin{aligned} \varPhi _{F2}&= \phi _{F2} \cdot (1 + \phi _{U2} + \phi _{U2}^2+ \cdots) = \dfrac{\phi _{F2}}{1-\phi _{U2}} \end{aligned}$$

where $\varPhi _{P2}$, $\varPhi _{N2}$, $\varPhi _{D2}$, and $\varPhi _{F2}$ are the overall yields of the whole bed of PS II units for photochemistry, regulated heat loss in the antennae, constitutive heat loss in the antennae, and fluorescence (mol energy dissipated $\hbox {mol}^{-1}$ energy absorbed).

Rate equations for steady-state electron transport through PS II can now be defined by combining Eqs. 25a, 16, and 15c. For PS II, electron transport depends on the balance between light absorption, excitation sharing, and regulated heat loss in the antennae (which control closure of open reaction centers), and Cyt $\hbox {b}_{6}\hbox {f}$ activity that is in excess of that supporting CEF1 (which controls re-opening of closed reaction centers). When an open PS II reaction center receives excitation, donates electrons to bound PQ, and accepts electrons from ${\hbox {H}_2\hbox {O}}$, it transitions to a closed state ($P680^0 \rightarrow P680^-$). The rate at which this occurs can be expressed as:

26a $$\begin{aligned} J_{P680}&= D_{P680}^0 \cdot k_{P680}^0 \end{aligned}$$

26b $$\begin{aligned} k_{P680}^0&= \left[ \dfrac{Q \cdot \alpha _2}{D^{}_{P680}} \cdot \left( \dfrac{1}{1-\phi _{U2}}\right) \right] \cdot \left( \dfrac{K_{P2}}{\varSigma K_{P680}^0}\right) \end{aligned}$$

where $D_{P680}^0$ is the concentration of open PS II centers ($\hbox {mol}\, \hbox {m}^{-2}$), $k_{P680}^0$ is a first-order turnover constant for open PS II centers ($\hbox {s}^{-1}$), and $\alpha _{2}$ is the absorbance cross-section associated with the PS II bed (mol PPFD absorbed by PS II $\hbox {mol}^{-1}$ incident PPFD). Analogously, closed centers re-open by exchanging bound $\hbox {PQH}_2$ for PQ ($P680^- \rightarrow P680^0$). The rate at which this occurs is given by:

27a $$\begin{aligned} J_{P680}&= D_{P680}^- \cdot k_{P680}^- \end{aligned}$$

27b $$\begin{aligned} k_{P680}^-&= \dfrac{D^{*}_{CB6F} \cdot k_{CB6F}^* }{D_{P680}^- \cdot \eta } \end{aligned}$$

where $D_{P680}^-$ is the concentration of closed PS II centers (mol $\hbox {m}^{-2}$), and $k_{P680}^-$ is a first-order turnover constant for closed PS II centers ($\hbox {s}^{-1}$). Mathematically, these expressions are different from those for PS I turnover in that the closing of open PS II centers is sensitive to the extent of excitation sharing and regulated heat loss within the PS II antennae, and the re-opening of closed centers is sensitive to the extent of CEF1. In the next section, we describe how the model can be solved by combining these rate equations for PS II, PS I, and Cyt $\hbox {b}_{6}\hbox {f}$ with the rate equations for Rubisco developed by Farquhar et al. (1980).

### Model solution

In this section, we describe how the equations in ‘Governing equations’ and ‘Electron transport rate equations’ are solved as a system. For the electron transport system to operate in a steady-state, the rates at which the populations of PS I and PS II in the open state transition into the closed state must be balanced by the rates at which the corresponding populations of complexes in the closed state transition back to the open state. At the same time, the development of the proton motive force must be balanced with its dissipation via the ATP synthase. In general, the electron and proton budgets can only be balanced when the electron transport system produces Fd, NADPH, and ATP at the same rates they are consumed by carbon metabolism. The solution to the model represents the idea that this steady-state balance is achieved by three regulatory interactions that bring all of the fluxes under the kinetic control of the most rate-limiting step in the system: photosynthetic control of Cyt $\hbox {b}_{6}\hbox {f}$, non-photochemical quenching of PS II, and cyclic electron flow around PS I (Fig. 1; red arrows). Specifically, we solve the model using three hypotheses: (i) LEF is always accompanied by at least enough CEF1 to balance the energy supply with the demands of carbon metabolism; (ii) NPQ functions to balance the excitation of PS II relative to that of PS I; and (iii) photosynthetic control functions to balance the activity of Cyt $\hbox {b}_{6}\hbox {f}$ relative to that of Rubisco.

#### Cyt $\hbox {b}_{6}\hbox {f}$-limited state

We use the term ‘Cyt $\hbox {b}_{6}\hbox {f}$-limited’ synonymously with the term ‘light-limited’ to refer to the metabolic state where electron transport is limiting carbon metabolism. Based on the PAM fluorescence analyses in Figs. 3 and 4, we posit that the Cyt $\hbox {b}_{6}\hbox {f}$-limited state is defined by two features of regulation. First, the system is poised in such a way that in the steady-state:

28 $$\begin{aligned} \dfrac{D^{-}_{P680}}{D^{}_{P680}} = \dfrac{D^{*}_{CB6F}}{D^{}_{CB6F}} = \dfrac{D^{+}_{P700}}{D^{}_{P700}} \end{aligned}$$

where the oxidation of the plastocyanin pool (i.e., equivalent to $D^{+}_{P700}/D^{}_{P700}$) is proportional to the reduction of the plastoquinone pool (i.e., equivalent to $D^{-}_{P680}/D^{}_{P680}$ and $D^{*}_{CB6F}/D^{}_{CB6F}$, as discussed in ‘Experiment’). Our interpretation is that this balance is achieved by regulation of the distribution of excitation to PS I and PS II (Fig. 1). Second, photosynthetic control is completely relaxed and the conductance of Cyt $\hbox {b}_{6}\hbox {f}$ is maximal:

29 $$\begin{aligned} k_{CB6F}^* = k_q \end{aligned}$$

such that $\hbox {PQH}_2$ is oxidized at the maximum potential rate. Combining Eqs. 28 and 29 with Eqs. 20a, 20b, 21a, and 21b yields expressions for the light-limited rates of electron transport:

30a $$\begin{aligned} J^{\prime }_{P700}&= \dfrac{V_{max\ (CB6F)} \cdot Q}{ \dfrac{V_{max\ (CB6F)}}{\alpha _1 \cdot K_{P1}/\varSigma K_{P700}^0 } + Q} \end{aligned}$$

30b $$\begin{aligned} J^{\prime }_{CB6F}&= J^{\prime }_{P700} \end{aligned}$$

30c $$\begin{aligned} J^{\prime }_{P680}&= J^{\prime }_{CB6F} \cdot \eta ^{-1} \end{aligned}$$

where $J^{\prime }_{P700}$, $J^{\prime }_{CB6F}$ and $J^{\prime }_{P680}$ are the rates of electron transport through PS I, Cyt $\hbox {b}_{6}\hbox {f}$, and PS II, respectively ($\hbox {mol}\,\hbox {e}^{-}\,\hbox {m}^{-2}\,\hbox {s}^{-1}$). In this state, the rate of net $\hbox {CO}_2$ assimilation is found by substituting Eq. 30c into Eq. 15a:

31 $$\begin{aligned} A_j&= \dfrac{J^{\prime }_{P680}}{4 + 8 \cdot \varGamma _{*}/C} \cdot \left( 1 - \varGamma _{*}/C \right) - R_d \end{aligned}$$

where $A_j$ is the potential rate of net $\hbox {CO}_2$ assimilation under Cyt $\hbox {b}_{6}\hbox {f}$ limitation.

#### Rubisco-limited state

We use the term ‘Rubisco-limited’ synonymously with the term ‘light-saturated’ to refer to the metabolic state where carbon metabolism is limiting electron transport. In this state, net $\hbox {CO}_2$ assimilation is given by the expression from Farquhar et al. (1980):

32 $$\begin{aligned} A_c&= \dfrac{V_{max \ (RUBC)} \cdot C}{K_c \cdot (1 + O/K_o) + C} \cdot \left( 1 - \varGamma _{*}/C \right) - R_d \end{aligned}$$

where $A_c$ is the potential rate of net $\hbox {CO}_2$ assimilation under Rubisco limitation and $V_{max \ (RUBC)}$ is the maximum carboxylase activity of Rubisco (mol $\hbox {CO}_2$ $\hbox {m}^{-2}$ $\hbox {s}^{-1}$). The corresponding rate of PS II electron transport can be derived by substituting Eq. 32 into Eq. 15a, but the rates of Cyt $\hbox {b}_{6}\hbox {f}$ and PS I electron transport depend on how photosynthetic control works. Specifically:

33a $$\begin{aligned}&J_{P680} = \dfrac{V_{max \ (RUBC)} \cdot C}{K_c \cdot (1 + O/K_o) + C} \cdot \left( 4 + 8 \cdot \varGamma _{*}/C\right) \end{aligned}$$

33b $$\begin{aligned}&J_{P680} \cdot \eta \le J_{CB6F} \le J^{\prime }_{CB6F} \end{aligned}$$

33c $$\begin{aligned}&J_{P700} = J_{CB6F} \end{aligned}$$

where the flux through Cyt $\hbox {b}_{6}\hbox {f}$ and PS I depends on whether there is a minimum CEF1 (with only an ATP-generating function), or a maximum CEF1 (with an additional regulatory function). Based on the PAM fluorescence analyses in Figs. 3 and 4, we posit that in either case:

34 $$\begin{aligned} \dfrac{D^{-}_{P680}}{D^{}_{P680}} = \dfrac{D^{*}_{CB6F}}{D^{}_{CB6F}} = \dfrac{Q}{ \dfrac{V_{max\ (CB6F)}}{\alpha _1 \cdot K_{P1}/\varSigma K_{P700}^0 } + Q} \end{aligned}$$

such that the PQ/$\hbox {PQH}_2$ pool always remains poised to maximize potential electron flow through Cyt $\hbox {b}_{6}\hbox {f}$ (n.b., Eq. 34 is derived from Eq. 30a). If there is only a minimum CEF1, then:

35 $$\begin{aligned} k_{CB6F}^* = k_q \cdot \dfrac{J_{CB6F}}{J^{\prime }_{CB6F}} \end{aligned}$$

such that intersystem electron transport is controlled at the sink-appropriate rate ($J_{CB6F} < J^{\prime }_{CB6F}$) by a reduction in the turnover constant of Cyt $\hbox {b}_{6}\hbox {f}$ ($k_{CB6F}^* < k_q$). If there is a maximum CEF1, Eq. 34 generalizes to Eq. 28, and 35 generalizes to Eq. 29.

#### Minimum of limiting rates

Under any given combination of environmental conditions (i.e., *Q*, $T_l$, *C*, *O*) and biochemical parameters (i.e., $\alpha _2$, $\alpha _1$, $V_{max\ (CB6F)}$, $V_{max\ (RUBC)}$), the actual rate of net $\hbox {CO}_2$ assimilation is given by:

36 $$\begin{aligned} A = \min \{A_j, A_c\} \end{aligned}$$

where $min\{\}$ represents the minimum of the potential limiting rates given in Eqs. 31 and 32 for $C > \varGamma _{*}$. In the Cyt $\hbox {b}_{6}\hbox {f}$-limited state, $A_j < A_c$ whereas in the Rubisco-limited state $A_c < A_j$. For each state, the equations in ‘[Sec Sec17]’ and ‘Rubisco-limited state’ can be used to derive the corresponding rates of electron transport ($J_{P680}$, $J_{P700}$), the photochemical yields ($\varPhi _{P2}$, $\varPhi _{P1}$), the degree of reaction center closure ($D^{-}_{P680}/D^{}_{P680}$, $D^{+}_{P700}/D^{}_{P700}$), and the turnover constant of Cyt $\hbox {b}_{6}\hbox {f}$ ($k_{CB6F}^*$). When the absorption cross-sections of PS II and PS I are specified, this system of equations can be solved to infer the rate constant for heat-dissipating forms of NPQ ($K_{N2}$). Alternatively, when the rate constant for heat-dissipating forms of NPQ is specified, this system of equations can also be solved to infer the absorption cross-sections of PS II and PS I ($\alpha _2$, $\alpha _1$). These solutions provide a basis for determining the overall yield of PS II for fluorescence emission, both in the steady-state and at the limits where all of the reaction centers are open or closed.

It is important to recognize that with this solution approach, the understanding of the limiting rates is being used to infer regulatory interactions from the ‘top down,’ i.e., starting from the observed functioning of the overall system and then decomposing this into sub-components. This is an unconventional strategy for modeling regulatory interactions like cyclic electron flow, non-photochemical quenching, and photosynthetic control. It may at first seem counterintuitive because it does not explicitly resolve the acidification of the thylakoid lumen, alkalinization of the stroma, and development of an electric field across the thylakoid membrane (e.g., Oja et al. 2011; Tikhonov 2013). These phenomena are often modeled with a ‘bottom-up’ approach that aims to piece together the detailed mechanisms that mediate the generation of the proton motive force, its partitioning into $\varDelta \psi$ and $\varDelta$pH, and the various responses to each of these signals (e.g., Davis et al. 2017; Lyu and Lazár 2017; Bennett et al. 2018). However, the ‘top down’ approach is an important complement to the ‘bottom-up’ approach because it facilitates a direct connection between the model and PAM fluorescence measurements and therefore allows for efficient evaluation of the hypotheses represented in the model. In the next section, we will demonstrate this principle with an inversion directly comparing the model to measurements from the sine wave experiment.

### Model inversion

In this section, we provide an example of how the model can be fit to PAM fluorescence and gas-exchange measurements, and how such fitting can be used both to interpret the measurements and to evaluate the model. We first develop coupling equations linking the model to PAM fluorescence and gas-exchange measurements; then describe a basic parameterization and an inversion framework based on multiobjective optimization; and finally present results of an inversion of measurements from the sine wave experiment.

#### Coupling expressions

In order to fit the model to PAM fluorescence and gas-exchange measurements, it is necessary to translate the model inputs and outputs into a form that is quantitatively consistent with the measurements. To link the model inputs to gas-exchange measurements, it is necessary to describe the diffusive path of $\hbox {CO}_2$ from the air surrounding a leaf into the sites of carboxylation in the chloroplasts. Here, we account for the diffusive resistances presented by the leaf boundary layer and stomata in the standard way, using measurements of the transpiration flux and leaf temperature. We then account for the diffusive resistance presented by mesophyll cell wall, cytosol, and chloroplast membrane with a single ‘mesophyll conductance’ term ($g_{m}$). While more complex formulations of mesophyll conductance have been proposed, we start with this because it provides the simplest way of translating between the quantity that is directly measured (i.e., partial pressure of $\hbox {CO}_2$ around the leaf) and the one that is needed to drive the model (i.e., partial pressure of $\hbox {CO}_2$ in the chloroplasts). To link the model outputs to PAM fluorescence, the approach is slightly more involved. At present, the conventions that are usually applied for interpreting PAM measurements are based on the assumption that all of the fluorescence reaching the detector is derived from PS II. With this approach, the steady-state fluorescence level is interpreted as:

37 $$\begin{aligned} \dfrac{F_s}{S} = \varPhi _{F2} \end{aligned}$$

where *S* is a factor representing the sensitivity of the optical detector to the steady-state fluorescence yield of PS II. However, it is also widely recognized that this convention does not support a truly quantitative analysis because the fluorescence signal reaching the PAM detectors includes light from PS I (e.g., Genty et al. 1990; Franck et al. 2002; Pfündel et al. 2013). With the model we have presented here, the total fluorescence level measured by a PAM detector can be interpreted as a sum of fluorescence fluxes derived from PS II and PS I:

38 $$\begin{aligned} \dfrac{F_s}{S} = \alpha _2 \cdot \varPhi _{F2} \cdot \varepsilon _{F2} + \alpha _1 \cdot \varPhi _{F1} \cdot \varepsilon _{F1} \end{aligned}$$

where each of the component fluxes depends on the corresponding absorption cross-section ($\alpha _2$, $\alpha _1$), fluorescence yield ($\varPhi _{F2}$, $\varPhi _{F1}$), and a weighting factor ($\varepsilon _{F2}$, $\varepsilon _{F1}$; mol fluorescent photons from PS II or PS I arriving at detector $\hbox {mol}^{-1}$ fluorescent photons emitted by PS II or PS I). With this approach, the maximum fluorescence level is given by:

39a $$\begin{aligned} \dfrac{F_m^{}}{S}&= \alpha _2 \cdot \left( \dfrac{K_{F2}}{\varSigma K_{P680}^{-}}\right) \cdot \varepsilon _{F2} + \alpha _1 \cdot \left( \dfrac{K_{F1}}{\varSigma K_{P700}^{+}}\right) \cdot \varepsilon _{F1} \end{aligned}$$

39b $$\begin{aligned} \varSigma K_{P680}^{-}&= K_{D2} + K_{F2}\end{aligned}$$

39c $$\begin{aligned} \varSigma K_{P700}^{+}&= K_{X1} + K_{D1} + K_{F1} \end{aligned}$$

in a dark-adapted leaf with all reaction centers closed, or by:

40a $$\begin{aligned} \dfrac{F_m^{'}}{S}&= \alpha _2 \cdot \left( \dfrac{K_{F2}}{\varSigma K_{P680}^{-}}\right) \cdot \varepsilon _{F2} + \alpha _1 \cdot \left( \dfrac{K_{F1}}{\varSigma K_{P700}^{+}}\right) \cdot \varepsilon _{F1} \end{aligned}$$

40b $$\begin{aligned} \varSigma K_{P680}^{-}&= K_{N2} + K_{D2} + K_{F2}\end{aligned}$$

40c $$\begin{aligned} \varSigma K_{P700}^{+}&= K_{X1} + K_{D1} + K_{F1} \end{aligned}$$

in a light-adapted leaf with all reaction centers closed. Analogously, the minimum fluorescence level is given by:

41a $$\begin{aligned} \dfrac{F_o^{}}{S}&= \alpha _2 \cdot \left( \dfrac{K_{F2}}{\varSigma K_{P680}^{0}}\right) \cdot \varepsilon _{F2} + \alpha _1 \cdot \left( \dfrac{K_{F1}}{\varSigma K_{P700}^{0}}\right) \cdot \varepsilon _{F1} \end{aligned}$$

41b $$\begin{aligned} \varSigma K_{P680}^{0}&= K_{P2} + K_{D2} + K_{F2}\end{aligned}$$

41c $$\begin{aligned} \varSigma K_{P700}^{0}&= K_{P1} + K_{D1} + K_{F1} \end{aligned}$$

in a dark-adapted leaf with all reaction centers open, or by:

42a $$\begin{aligned} \dfrac{F_o^{'}}{S}&= \alpha _2 \cdot \left( \dfrac{K_{F2}}{\varSigma K_{P680}^{0}}\right) \cdot \varepsilon _{F2} + \alpha _1 \cdot \left( \dfrac{K_{F1}}{\varSigma K_{P700}^{0}}\right) \cdot \varepsilon _{F1} \end{aligned}$$

42b $$\begin{aligned} \varSigma K_{P680}^{0}&= K_{P2} + K_{N2} + K_{D2} + K_{F2}\end{aligned}$$

42c $$\begin{aligned} \varSigma K_{P700}^{0}&= K_{P1} + K_{D1} + K_{F1} \end{aligned}$$

in a light-adapted leaf with all reaction centers open. These expressions can be used to model any PAM measurements by adjusting the weighting factors ($\varepsilon _{F1}, \varepsilon _{F2}$) to account for the emission spectra of PS I and PS II, the escape ratio of fluorescence as a function of wavelength, and the spectral response of a given PAM detector. The modeled $F_s^{}$, $F_m^{}$, $F_m^{'}$, $F_o^{}$, and $F_o^{'}$ values can then be combined to calculate the ‘apparent’ values of any of the ratio-based indices that are commonly derived from PAM measurements.

#### Variable selection and parameterization

To operationalize the expressions above in an inversion, the next step is to specify which model parameters to constrain with experimental measurements (inputs), and which to treat as free variables (outputs). For this analysis, we have constrained as many parameters as possible with experimental measurements, either directly from the sine wave experiment or from the literature. These are summarized in Table 1 and discussed below.

**Table 1:** Input parameters for inversions

Category	Symbol	Value(s)	Units	Description
Environmental variables	*Q*	0.1 to 2293.5	umol PPFD $\hbox {m}^{-2}\,\hbox {s}^{-1}$	Photosynthetically active radiation
	*T*	$25.01 \pm 0.01$	$^{\circ }\hbox {C}$	Leaf temperature
	*C*	$407.0 \pm 0.4$	$\upmu$bar $\hbox {CO}_2$	Partial pressure of $\hbox {CO}_2$ in cuvette
	*O*	$212.7 \pm 0.1$	mbar $\hbox {O}_2$	Partial pressure of $\hbox {O}_2$ in cuvette
	*P*	$1.017 \pm 0.001$	bar	Total pressure in cuvette
Physiological variables	$\alpha$	$0.832 \pm 0.003$	$\hbox {mol}\,\hbox {mol}^{-1}$	Total leaf absorbance to PAR
	$F_s^{}$	747 to 963	dimensionless	Steady-state fluorescence
	$F_m^{}$, $F_m^{'}$	824 to 3905	dimensionless	Maximum fluorescence in the dark and in the light
	$F_o^{}$, $F_o^{'}$	568 to 797	dimensionless	Minimum fluorescence in the dark and in the light
	*A*	− 0.7 to 12.2	$\upmu \hbox {mol}\,\hbox {CO}_2\,\hbox {m}^{-2}\,\hbox {s}^{-1}$	Net $\hbox {CO}_2$ assimilation rate
	$g_{tc}$	0.01 to 0.09	$\hbox {mol} \,\hbox {CO}_2\,\hbox {m}^{-2}\,\hbox {s}^{-1}$	Total (stomatal and boundary layer) conductance to $\hbox {CO}_2$
	*E*	0.5 to 2.3	$\hbox {mmol} \,\hbox {H}_2\hbox {O}\,\hbox {m}^{-2}\,\hbox {s}^{-1}$	Transpiration rate
Photochemical constants	$K_{F1}$, $K_{F2}$	0.05	$\hbox {ns}^{-1}$	Rate constant for fluorescence at PS I & PS II
	$K_{D1}$, $K_{D2}$	0.55	$\hbox {ns}^{-1}$	Rate constant for constitutive heat loss at PS I & PS II
	$K_{P1}$	14.5	$\hbox {ns}^{-1}$	Rate constant for photochemistry at PS I
	$K_{P2}$	4.5	$\hbox {ns}^{-1}$	Rate constant for photochemistry at PS II
Biochemical constants	$k_q$	300	mol $\hbox {PQH}_2$ $\hbox {mol}^{-1}$ sites $\hbox {s}^{-1}$	Catalytic constant for $\hbox {PQH}_2$ for Cyt $\hbox {b}_{6}\hbox {f}$
	$n_L$	0.75	mol ATP $\hbox {mol}^{-1}$ e$^-$	Coupling efficiency of linear electron flow
	$n_C$	1.00	mol ATP $\hbox {mol}^{-1}$ e$^-$	Coupling efficiency of cyclic electron flow
	$k_c$	3.6	mol $\hbox {CO}_2$ $\hbox {mol}^{-1}$ sites $\hbox {s}^{-1}$	Catalytic constant for $\hbox {CO}_2$ for Rubisco
	$k_o$	0.9	mol $\hbox {O}_2$ $\hbox {mol}^{-1}$ sites $\hbox {s}^{-1}$	Catalytic constant for $\hbox {O}_2$ for Rubisco
	$K_c$	260	$\upmu$bar	Michaelis constant for $\hbox {CO}_2$ for Rubisco
	$K_o$	179	mbar	Michaelis constant for $\hbox {O}_2$ for Rubisco

For the inversions, all of the listed environmental and physiological parameters are measured variables. The constants are all derived from literature values, and correspond to a reference temperature of 25 $^{\circ }\hbox {C}$. See ‘Variable selection and parameterization’ for details

##### Environmental and physiological variables

The driving environmental variables are measured values of light intensity (*Q*), leaf temperature (*T*), cuvette $\hbox {CO}_2$ and $\hbox {O}_2$ partial pressure (*C*, *O*), and total pressure in the cuvette (*P*). The physiological variables are measured values of the total leaf absorbance to PAR ($\alpha$), the steady-state fluorescence levels in the light ($F_s$), the maximum fluorescence levels in the dark and light ($F_m^{}$ and $F_m^{'}$), the minimum fluorescence levels in the dark and light ($F_o^{}$ and $F_o^{'}$), the net $\hbox {CO}_2$ assimilation rate (*A*), total conductance to $\hbox {CO}_2$ ($g_{tc}$), and transpiration rate (*E*). The total leaf absorbance to PAR was measured with an integrating sphere (Analytical Spectral Devices, Inc.) and spectrometer (AvaSpec-ULS3648, Avantes), and the fluorescence and gas-exchange were measured as described in ‘Experiment’.

##### Photochemical constants

To parameterize the PS I and PS II rate constants for photochemistry ($K_{P1}$, $K_{P2}$), constitutive heat dissipation ($K_{D1}$, $K_{D2}$), and fluorescence ($K_{F1}$, $K_{F2}$), we utilize fluorescence lifetime measurements from higher plants (e.g., see review by Chukhutsina et al. 2018). The rate constants for fluorescence are specified to have an absolute value of 0.05 n$\hbox {s}^{-1}$, and the other rate constants are scaled relative to this value using measurements summarized in Wientjes et al. (2017). For PS I, we specify a scaling that translates to a maximum photochemical yield of $96\%$, average fluorescence lifetime of 65 ps, and fluorescence yield of $0.35\%$. For PS II, we specify a scaling that translates to a maximum photochemical yield of $88\%$, average fluorescence lifetimes of 200 ps ($F_o^{}$) to 1.6 ns ($F_m^{}$), and fluorescence yields of $1\%$ ($F_o^{}$) to $8\%$ ($F_m^{}$). For PS I, $K_{X1}$ represents heat loss via oxidized PS I centers. We specify that $K_{X1}$ is numerically equivalent to $K_{P1}$, such that closed PS I centers quench excitation to heat as efficiently as open PS I centers quench excitation photochemically.

##### Biochemical constants

To parameterize the maximum potential turnover rate of Cyt $\hbox {b}_{6}\hbox {f}$ ($k_q$), we use a value of 300 mol $\hbox {PQH}_2$ $\hbox {mol}^{-1}$ sites $\hbox {s}^{-1}$ which corresponds to the low end of the range of in vitro estimates that correspond to this state in higher plants (Dietrich and Kühlbrandt 1999; Zhang et al. 2001) and the high end of the range of in vivo estimates (Laisk and Oja 1994, 1995; Laisk et al. 2005a, 2016). We then link electron flow to the proton circuit via composite coupling efficiencies. For LEF and CEF1, $n_L$ and $n_C$ are assigned values of 0.75 and 1.00 mol ATP $\hbox {mol}^{-1}$ electrons, respectively. These values assume that the Cyt $\hbox {b}_{6}\hbox {f}$ has a constitutive Q-cycle (2 $\hbox {H}^{+}$/$\hbox {e}^{-}$) (Sacksteder et al. 2000), all protons pumped into the thylakoid pass out through the ATP synthase, and the ATP synthase operates at the thermodynamic stoichiometry (4 $\hbox {H}^{+}$/ATP) (Petersen et al. 2012). For $n_C$, the specified value also assumes that electrons are transferred only via the NADH dehydrogenase-like complex (NDH) which serves as a proton pump (2 $\hbox {H}^{+}$/$\hbox {e}^{-}$) (Strand et al. 2017). Finally, the definition and parameterization of the rate constants for Rubisco are as described by von Caemmerer et al. (2009) (i.e., see Table 9.1 in that reference, values at $25^{\circ }$C, scaled for finite mesophyll conductance).

#### Fitting strategy, fitted parameters, and fit quality

After assigning the inputs above, the following parameters remain as free variables: the relative absorption cross-sections of PS II and PS I ($\alpha _2$, $\alpha _1$), the rate constant for excitation sharing within the PS II antennae ($K_{U2}$), the maximum activity of Cyt $\hbox {b}_{6}\hbox {f}$ ($V_{max\ (CB6F)}$), the mesophyll conductance to $\hbox {CO}_2$ ($g_m$), the maximum activity of Rubisco ($V_{max\ (RUB)}$), and the relative weighting of PS I versus PS II fluorescence in the PAM signal ($\varepsilon _{F1}/\varepsilon _{F2}$). We have estimated these variables using measurements from a single leaf of *P. fremontii* over the ascending phase of the highest light intensity treatment in the sine wave experiment. For this analysis, the biochemical model was configured to permit state transitions under limiting light and to hold the pigment distribution constant under saturating light. The modeled values of the absorption cross-sections and fluorescence yields were combined to predict the observed values of the PAM measurements using the coupling expressions above. The model was then fit to the measurements using a multiple objective optimization procedure implemented with a genetic algorithm. The objective function simultaneously minimized the differences between measured and modeled values of PAM fluorescence and $\hbox {CO}_2$ exchange. Tests with synthetic data that mimicked the real sampling design and error characteristics demonstrated that this procedure could be expected to retrieve the free parameters to within +/-1% of their true values.

The parameter estimates from this optimization procedure represent a population of equivalent solutions on a Pareto front. As this population is not always normally distributed, we report the parameter estimates in terms of medians and interquartile ranges, i.e., 50th (25th, 75th). The rate constant for excitation sharing in the PS II antennae was estimated to be 2.1 (1.3, 2.8) $\hbox {ns}^{-1}$. This was estimated to drive 13 (10, 16) % decreases in the PS II cross-section and complementary increases in the PS I cross-section between complete darkness and the light saturation point. The maximum activities of Cyt $\hbox {b}_{6}\hbox {f}$ and Rubisco were estimated to be 378 (357, 441) $\upmu$mol e$^-$ $\hbox {m}^{-2}$ $\hbox {s}^{-1}$ and 114 (106, 120) $\upmu$mol $\hbox {CO}_2$ $\hbox {m}^{-2}$ $\hbox {s}^{-1}$. The mesophyll conductance to $\hbox {CO}_2$ was estimated to be 0.084 (0.073, 0.098) mol $\hbox {CO}_2$ $\hbox {m}^{-2}$ $\hbox {s}^{-1}$ bar$^{-1}$. The relative weighting of PS I versus PS II fluorescence in the PAM signal was estimated to be 2.0 (1.6, 2.5) mol PS I $\hbox {mol}^{-1}$ PS II. This translates to PS I contributing 39% and 7% of the total fluorescence signal at $F_o$ and $F_m$, respectively.

**Fig. 5:**
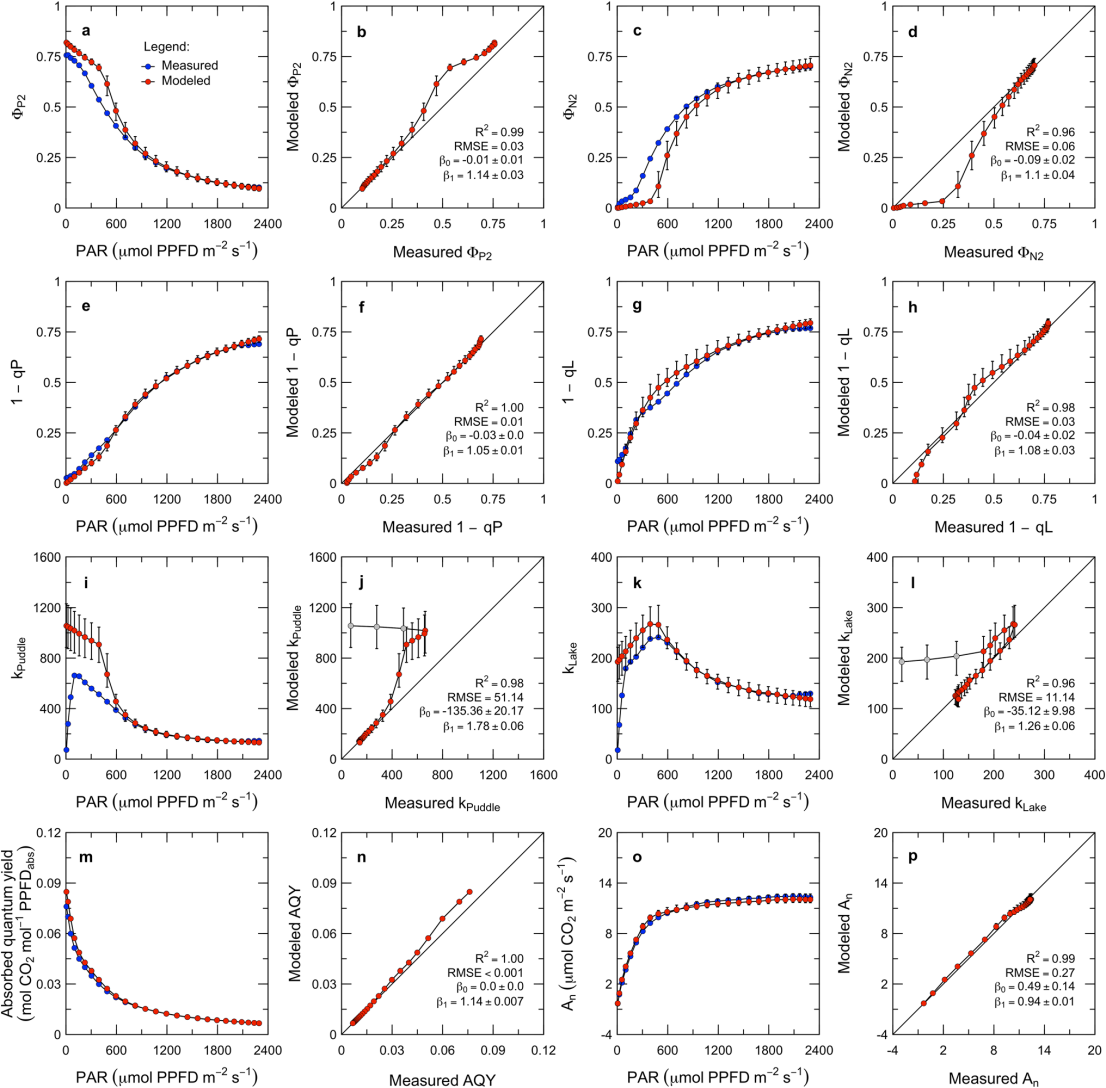
Example of model inversion with measurements from sine wave experiment. These plots illustrate the fit of the model to fluorescence and gas-exchange measurements of a single leaf of *P. fremontii* over the ascending phase of the highest light intensity treatment in the sine wave experiment. For each parameter, the modeled and measured values are illustrated as a function of light intensity, and relative to an ideal 1:1 relationship. The modeled values are given as the median and interquartile range across a set of simulations based on a population of Pareto optimal parameter estimates. The quality of fit is assessed with Type I regressions and summarized with the coefficient of determination ($\hbox {R}^{2}$), root mean square error (RMSE), and intercept and slope ($\beta _0$, $\beta _1$). Outliers shaded in gray are excluded from the quality-of-fit statistics. Note that the apparent redox state of the PQ pool is given both as $1 - qP$ (Schreiber et al. 1986) and $1 - qL$ (Kramer et al. 2004). Analogously, the apparent conductance of Cyt $\hbox {b}_{6}\hbox {f}$ is given both as $k_{Puddle} = LEF/(1 - qP)$ and $k_{Lake} = LEF/(1 - qL)$. The input parameters are given in Table 1, and other details as described in ‘Model inversion’

With this optimized parameterization, the model is capable of explaining the vast majority of the variation in both the PAM fluorescence and the gas-exchange measurements (i.e., average $\hbox {R}^{2} >98\%$; Fig. 5). In general, the skill of the model is highest under conditions that are the typical for the Cyt $\hbox {b}_{6}\hbox {f}$-limited and Rubisco-limited states, and reduced under conditions associated with transitions into or out of these states. For example, there is systematic divergence between measured and modeled values around the light saturation point (Fig. 5a–d), as well as at the limit where light goes to zero (Fig. 5i–l). On the one hand, this pattern provides support for the quantitative description that we have proposed for the Cyt $\hbox {b}_{6}\hbox {f}$-limited and Rubisco-limited states. On the other hand, it also indicates that there are structural errors in the model formulation, and suggests that these errors are related to the quantitative description of the regulatory processes that mediate the transitions between the Cyt $\hbox {b}_{6}\hbox {f}$-limited and Rubisco-limited states.

### Model simulations

In this section, we present a series of model simulations. The simulations each perturb a different aspect of the structure and/or regulation of electron transport and analyze the model’s response. Cases 1–4 address the factors that control photosynthesis in the Cyt $\hbox {b}_{6}\hbox {f}$-limited state; Cases 5–8 address how photosynthesis transitions between the Cyt $\hbox {b}_{6}\hbox {f}$-limited and Rubisco-limited states; and Cases 9-12 address how the interactions between electron transport and carbon metabolism are expressed in observable gas-exchange and fluorescence parameters. The parameterization for the base case was designed to approximate the sine wave experiment, and is summarized in Table 2.

**Table 2:** Input parameters for simulations

Category	Symbol	Value(s)	Units	Description
Environmental variables	*Q*	0 to 2400	umol PPFD $\hbox {m}^{-2}\,\hbox {s}^{-1}$	Photosynthetically active radiation
	*T*	25	$^{\circ }$C	Leaf temperature
	*C*	200	$\upmu$bar $\hbox {CO}_2$	Partial pressure of $\hbox {CO}_2$ in chloroplast
	*O*	209	mbar $\hbox {O}_2$	Partial pressure of $\hbox {O}_2$ in chloroplast
Physiological variables	$\alpha$	0.85	mol $\hbox {mol}^{-1}$	Total leaf absorbance to PAR
	$\alpha _{1}$, $\alpha _{2}$	0.41, 0.44	mol $\hbox {mol}^{-1}$	Absorbance cross-section of PS I & PS II
	$V_{max\ (CB6F)}$	350	$\upmu$mol e$^-$ $\hbox {m}^{-2}\,\hbox {s}^{-1}$	Maximum activity of Cyt $\hbox {b}_{6}\hbox {f}$
	$V_{max\ (RUBC)}$	100	$\upmu$mol $\hbox {CO}_2\,\hbox {m}^{-2}$ $\hbox {s}^{-1}$	Maximum carboxylase activity of Rubisco
	$R_d$	0.01	%	Dark respiration scaled to ${V}_{\mathrm{max}}$ Rubisco
Photochemical constants	$K_{F1}$, $K_{F2}$	0.05	$\hbox {ns}^{-1}$	Rate constant for fluorescence at PS I & PS II
	$K_{D1}$, $K_{D2}$	0.55	$\hbox {ns}^{-1}$	Rate constant for constitutive heat loss at PS I & PS II
	$K_{P1}$	14.5	$\hbox {ns}^{-1}$	Rate constant for photochemistry at PS I
	$K_{P2}$	4.5	$\hbox {ns}^{-1}$	Rate constant for photochemistry at PS II
	$K_{U2}$	0	$\hbox {ns}^{-1}$	Rate constant for excitation sharing at PS II
Biochemical constants	$k_q$	300	mol $\hbox {PQH}_2\,\hbox {mol}^{-1}$ sites $\hbox {s}^{-1}$	Catalytic constant for $\hbox {PQH}_2$ for Cyt $\hbox {b}_{6}\hbox {f}$
	$n_L$	0.75	mol ATP $\hbox {mol}^{-1}$ e$^-$	Coupling efficiency of linear electron flow
	$n_C$	1.00	mol ATP $\hbox {mol}^{-1}$ e$^-$	Coupling efficiency of cyclic electron flow
	$k_c$	3.6	mol $\hbox {CO}_2\,\hbox {mol}^{-1}$ sites $\hbox {s}^{-1}$	Catalytic constant for $\hbox {CO}_2$ for Rubisco
	$k_o$	0.9	mol $\hbox {O}_2\,\hbox {mol}^{-1}$ sites $\hbox {s}^{-1}$	Catalytic constant for $\hbox {O}_2$ for Rubisco
	$K_c$	260	$\upmu$bar	Michaelis constant for $\hbox {CO}_2$ for Rubisco
	$K_o$	179	mbar	Michaelis constant for $\hbox {O}_2$ for Rubisco

For the simulations, all of the input parameters are specified to approximate the sine wave experiment. See ‘Variable selection and parameterization’ for details

#### Limits of electron transport (Cases 1–4)

**Fig. 6:**
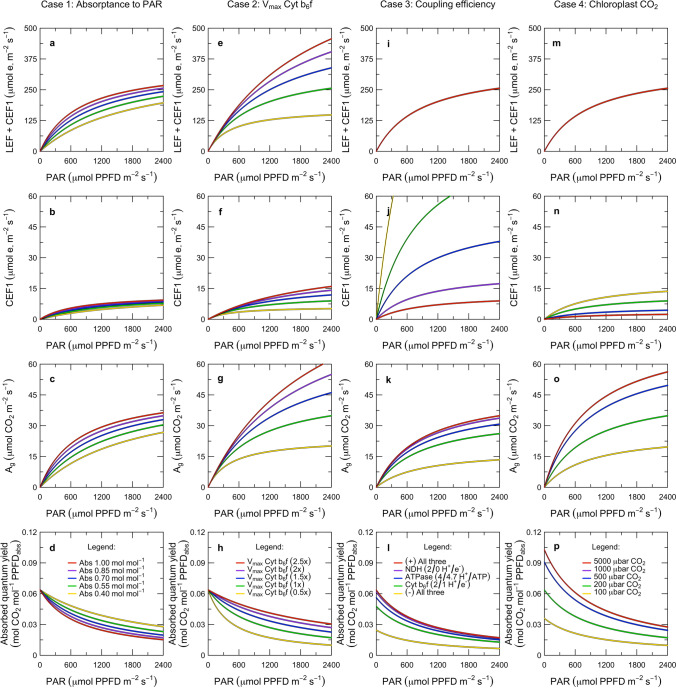
Key model predictions related to the structure of the electron transport system. These simulations illustrate the effects of variation in parameters that control electron transport under limiting light intensities: the absorptance to PAR (Case 1), the maximum activity of Cyt $\hbox {b}_{6}\hbox {f}$ (Case 2), the efficiency of coupling between electron transport and ATP production (Case 3), and the chloroplast $\hbox {CO}_2$ (Case 4). Results are plotted for the potential rates of LEF and CEF1 together, the potential rate of CEF1 alone, the potential rate of gross $\hbox {CO}_2$ assimilation, and the potential absorbed quantum yield for $\hbox {CO}_2$ assimilation. Note that the rates are described as ‘potential’ because they all correspond to the Cyt $\hbox {b}_{6}\hbox {f}$-limited state. The input parameters are given in Table 2, and other details as described in ‘Limits of electron transport (Cases 1–4)’

The first set of cases addresses the Cyt $\hbox {b}_{6}\hbox {f}$-limited state. In Fig. 6, all of the corresponding simulations show the full ‘potential’ value of each parameter in the Cyt $\hbox {b}_{6}\hbox {f}$-limited state, i.e., without considering the transition to the Rubisco-limited state, or as if the activity of Rubisco was high enough not to impose such a transition. In all of these simulations, there is no connectivity between PS II units, and the pigment system is immobile.

The primary environmental factor that limits the potential rate of electron transport is the availability of light, and this is modulated by leaf absorptance to PAR (Fig. 6, Case 1). Under limiting light intensities, the potential rate of electron flow through Cyt $\hbox {b}_{6}\hbox {f}$ and PS I is a rectangular hyperbolic function of light intensity (Fig. 6a). The geometry of this function demonstrates that light drives a trade-off between the speed and efficiency of electron transport: the increase in speed is caused by the increased supply of reduced plastoquinone to Cyt $\hbox {b}_{6}\hbox {f}$, and the decrease in efficiency is caused by the simultaneous accumulation of closed reaction centers. In these simulations, the PAR absorptance has been varied while maintaining a constant partitioning of absorbed light between PS I and PS II. The resulting variation in the amount of light reaching PS I and PS II alters the initial slope and apparent curvature of the light response, i.e., the half-saturation point of Eq. 30a (Fig. 6a). The variation in the potential rates of LEF and CEF1 does not influence the partitioning between LEF and CEF1 (Fig. 6b), but does drive variation in the potential rates of gross $\hbox {CO}_2$ assimilation as well as the absorbed quantum yield for $\hbox {CO}_2$ assimilation (Fig. 6c, d). Increasing the absorptance to PAR drives simultaneous increases in the rates of photosynthesis and decreases in the absorbed quantum yields. The basis of these effects is that increasing the absorptance to PAR increases the rate at which open reaction centers are driven to the closed state, and therefore increases the accumulation of closed reaction centers.

The primary biochemical factor that limits the potential rate of electron transport is the maximum activity of Cyt $\hbox {b}_{6}\hbox {f}$ (Fig. 6, Case 2). In Eq. 30a, the final asymptote is set by the maximum activity of Cyt $\hbox {b}_{6}\hbox {f}$. Although Cyt $\hbox {b}_{6}\hbox {f}$ operates well below this upper limit in these simulations, the limit nevertheless structures the potential rate of electron transport across the full range of natural light intensities (Fig. 6e). Variation in the maximum activity of Cyt $\hbox {b}_{6}\hbox {f}$ causes variation in the potential rates of LEF and CEF1, but CEF1 always represents a small and constant fraction of LEF (Fig. 6f). The variation in the potential rates of LEF and CEF1 then causes variation in the potential rates of gross $\hbox {CO}_2$ assimilation as well as the absorbed quantum yield for $\hbox {CO}_2$ assimilation (Fig. 6g, h). In contrast to Case 1, increasing the maximum activity of Cyt $\hbox {b}_{6}\hbox {f}$ enhances the rates of photosynthesis as well as the absorbed quantum yields over the range of natural light intensities. The basis of these effects is that, at any given excitation pressure on PS I and PS II, increasing the maximum activity of Cyt $\hbox {b}_{6}\hbox {f}$ increases the rate at which closed reaction centers re-open and therefore decreases the accumulation of closed reaction centers. This has the effect of modulating the light-driven trade-off between the speed and efficiency of electron transport.

At a given potential rate of electron transport, the efficiency of coupling between electron flow and the proton circuit presents a third biochemical limitation to photosynthesis (Fig. 6, Case 3). The reference parameterization describes a maximum efficiency scenario in which: (1) the Cyt $\hbox {b}_{6}\hbox {f}$ Q-cycle operates constitutively in LEF as well as CEF1, (2) the NDH complex functions as a high efficiency proton pump in CEF1, and (3) the ATP synthase operates at the thermodynamic stoichiometry rather than the structural stoichiometry. With these assumptions in place, CEF1 represents only 3% of total electron flow. While relaxing these assumptions has no effect on the potential rate of electron flow (Fig. 6i), it increases the fraction of total electron flow through CEF1 versus LEF (Fig. 6j). Omitting the proton pumping associated with NDH increases CEF1 to 6% of the total electron flow; assuming the ATP synthase operates at the structural stoichiometry increases CEF1 to 14%; omitting the Cyt $\hbox {b}_{6}\hbox {f}$ Q-cycle increases CEF1 to 27%; and relaxing all three assumptions simultaneously increases CEF1 to 63%. At any given light intensity, the associated decreases in LEF limit the potential rates of gross $\hbox {CO}_2$ assimilation as well as the absorbed quantum yield for $\hbox {CO}_2$ assimilation (Fig. 6k, l).

Analogously, the demands of carbon metabolism for Fd, NADPH, and ATP present a fourth biochemical limitation to photosynthesis (Fig. 6, Case 4). In these simulations, the activities of the PCR and PCO cycles have been varied by manipulating the partial pressure of chloroplast $\hbox {CO}_2$ at a constant partial pressure of $\hbox {O}_2$. While increasing the $\hbox {CO}_2$/$\hbox {O}_2$ ratio has no effect on the potential rate of electron flow (Fig. 6m), it decreases the fraction of total electron flow through CEF1 versus LEF in order to support increases in PCR versus PCO cycle activity (Fig. 6n). This enhances the potential rates of gross $\hbox {CO}_2$ assimilation as well as the absorbed quantum yield for $\hbox {CO}_2$ assimilation (Fig. 6o, p). Due to the high activity of the PCO cycle relative to the PCR cycle at 200 $\upmu$bar chloroplast $\hbox {CO}_2$, the maximum absorbed quantum yield that is expressed is $0.064 \,\hbox {mol} \,\hbox {CO}_2\,\hbox {mol}^{-1}$ absorbed PPFD (Fig. 6p; green line). All else being equal, suppressing PCO cycle activity by increasing $\hbox {CO}_2$ to 5,000 $\upmu$bar increases the maximum absorbed quantum yield to $0.103 \,\hbox {mol} \,\hbox {CO}_2\,\hbox {mol}^{-1}$ absorbed PPFD (Fig. 6p; red line).

#### Regulation of electron transport (Cases 5–8)

**Fig. 7:**
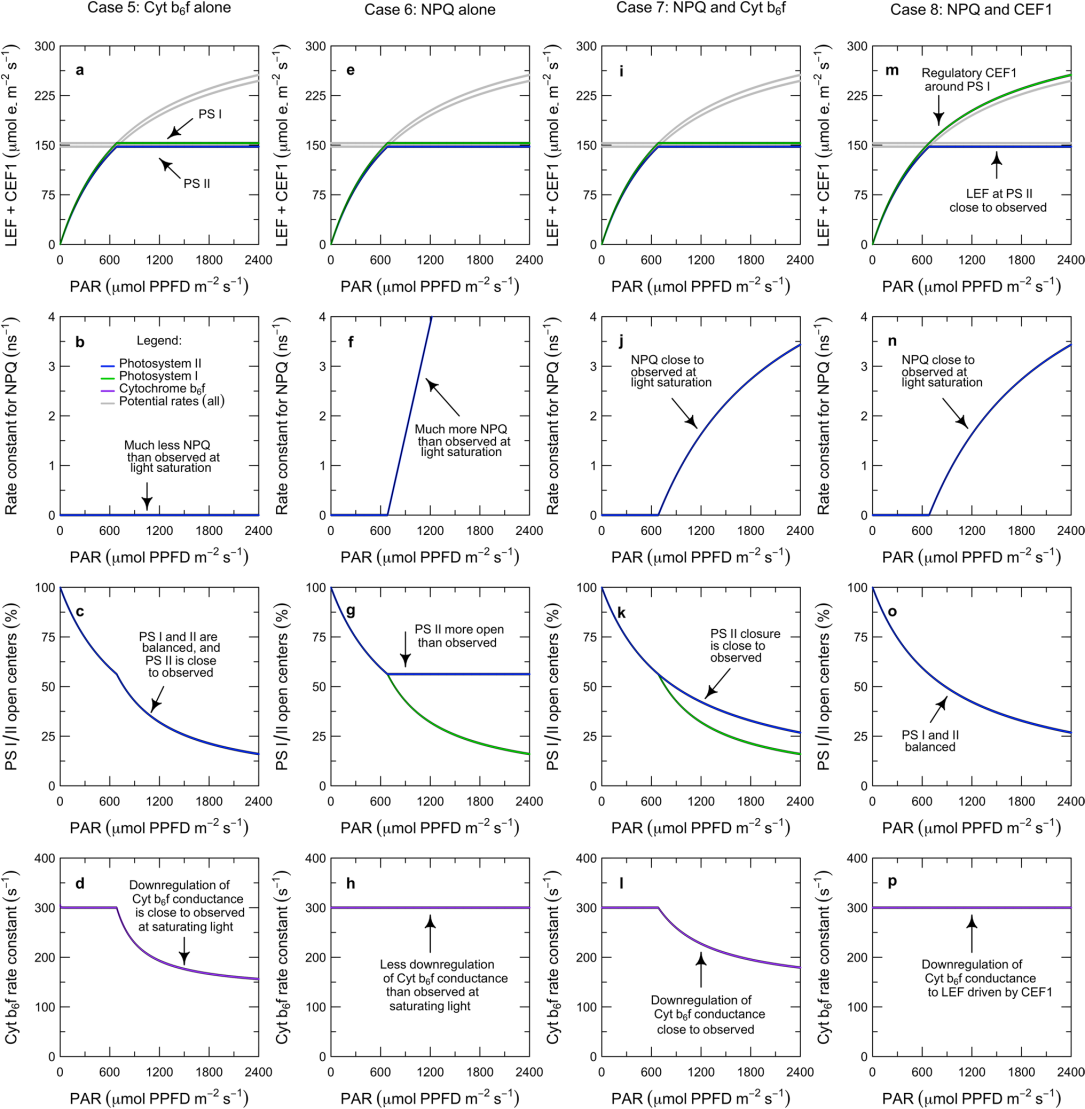
Key model predictions related to the regulation of the electron transport system. These simulations examine the effects of variation in assumptions about how electron transport is coordinated with carbon metabolism: via feedback regulation of Cyt $\hbox {b}_{6}\hbox {f}$ alone (Case 5), via feedback regulation of NPQ alone (Case 6), via feedback regulation of NPQ and Cyt $\hbox {b}_{6}\hbox {f}$ together (Case 7), and via feedback regulation of NPQ and CEF1 together (Case 8). Results are plotted for the potential and actual rates of LEF and CEF1, the rate constant for NPQ, the fraction of open reaction centers for PS II and PS I, and the rate constant for Cyt $\hbox {b}_{6}\hbox {f}$. The input parameters are given in Table 2, and other details are as described in ‘Regulation of electron transport (Cases 5–8)’

We now turn to the question of how regulation coordinates transitions between the Cyt $\hbox {b}_{6}\hbox {f}$-limited and Rubisco-limited states. In Fig. 7, the potential rates of electron transport are plotted in gray for both the Cyt $\hbox {b}_{6}\hbox {f}$-limited and Rubisco-limited states. Under any specific condition, the minimum of the potential rates corresponds to the actual rate, and is plotted in color. Note that for simplicity we have again specified that there is no connectivity between PS II units, and that the pigment system is immobile.

We begin with a simulation in which photosynthetic control is achieved purely via feedback on Cyt $\hbox {b}_{6}\hbox {f}$ (Fig. 7, Case 5). In this simulation, feedback acts on Cyt $\hbox {b}_{6}\hbox {f}$ alone to balance the rates of LEF and CEF1 with the activities of the PCR and PCO cycles (Fig. 7a). It is prescribed that there is no development of heat-dissipating forms of NPQ (Fig. 7b). Due to the fluorescence lifetime parameterization, the maximum photochemical yield of PS I ($96\%$) is slightly higher than that of PS II ($88\%$). In addition, due to the environmental conditions, PCO cycle activity requires a low level of CEF1. However, the PS II and PS I absorption cross-sections are specified in a way that compensates for these supply and demand effects and balances the excitation of PS II and PS I (Fig. 7c). Below the light saturation point, Cyt $\hbox {b}_{6}\hbox {f}$ turns over at the maximum rate, and above the light saturation point, feedback slows the turnover of Cyt $\hbox {b}_{6}\hbox {f}$ to limit electron transport through PS II and PS I to a rate that remains in balance with the capacity of carbon metabolism (Fig. 7d).

Next, we examine a simulation in which photosynthetic control is achieved purely via feedback on NPQ (Fig. 7, Case 6). In this simulation, feedback acts on NPQ alone to balance the rates of LEF and CEF1 to the activities of the PCR and PCO cycles (Fig. 7e). There is no NPQ under limiting light and a strong acceleration in the rate of increase in NPQ above the light saturation point (Fig. 7f). The absence of NPQ under limiting light intensities reflects the fact that the absorption cross-sections are balanced and there is no connectivity prescribed for the PS II antennae. As a result, the closure of PS I and PS II remains balanced under limiting light (Fig. 7g). At and above the light saturation point, the increase in NPQ poises the plastoquinone pool at a point where the rate of electron flow through PS II remains in balance with the excitation of PS I without any further feedback on Cyt $\hbox {b}_{6}\hbox {f}$ (Fig. 7g, h). However, neither this scenario nor the previous scenario are consistent with the experimental observations at saturating light in Figs. 2h and 3c.

A scenario that is more consistent with the sine wave experiment is that photosynthetic control is achieved by an interaction between NPQ and Cyt $\hbox {b}_{6}\hbox {f}$ (Fig. 7, Case 7). Under limiting light, the dynamics of this scenario are equivalent to those of the previous scenario: there is no NPQ, the closure of PS II is balanced with that of PS I, and the rate constant for $\hbox {PQH}_2$ oxidation at Cyt $\hbox {b}_{6}\hbox {f}$ remains at its maximum value (Fig. 7i–l). Under saturating light, there is still an acceleration in the rate of increase in NPQ (Fig. 7j). However, it is not as dramatic as in the previous scenario because a simultaneous feedback slows Cyt $\hbox {b}_{6}\hbox {f}$ turnover (Fig. 7l). The level of NPQ maintains the plastoquinone pool at the redox poise that corresponds to the Cyt $\hbox {b}_{6}\hbox {f}$-limited state (Fig. 7k). The simultaneous decrease in the apparent conductance of Cyt $\hbox {b}_{6}\hbox {f}$ then limits LEF and CEF1 to rates that are balanced with the capacity of the PCR and PCO cycles to consume Fd, NADPH, and ATP (Fig. 7i).

Increased flux through CEF1 upon reaching light saturation has been proposed as another way to match LEF to sink capacity (Fig. 7, Case 8). For example, electron transport through Cyt $\hbox {b}_{6}\hbox {f}$ could in principle be controlled at a sink-appropriate rate with a regulatory CEF1 flux above the light saturation point (e.g., Heber and Walker 1992) (Fig. 7m). By ‘regulatory CEF1 flux,’ we mean one that is somehow uncoupled from the ATP demands of carbon metabolism (e.g., via a decrease in proton influx into the lumen, or an increase in proton efflux from the lumen through a pathway other than the ATP synthase). If photosynthetic control of Cyt $\hbox {b}_{6}\hbox {f}$ were achieved in this way, then the closure of PS I and PS II could remain exactly balanced (Fig. 7o) and Cyt $\hbox {b}_{6}\hbox {f}$ could remain at its maximum turnover constant (Fig. 7p). Such a scenario would imply that Eqs. 28 and 29 hold across the entire range of light intensities. While both Case 7 and Case 8 are consistent with our experimental observations (Figs. 3 and 4), the latter could also help to explain the relationship between NPQ and the poise of the plastoquinone pool under saturating light (Fig. 7n, o).

#### Parameterization of the model (Cases 9–12)

In the previous case studies we have illustrated some of the basic properties of the Cyt $\hbox {b}_{6}\hbox {f}$-limited state, and examined possible ways for representing the transition from the Cyt $\hbox {b}_{6}\hbox {f}$-limited state to the Rubisco-limited state. For the cases in this section, we present full simulations that include the transition from the Cyt $\hbox {b}_{6}\hbox {f}$-limited state to the Rubisco-limited state. In these simulations, we examine the effects of two key aspects of the model parameterization: the interaction between the connectivity of the PS II antennae and the redistribution of pigment between PS II and PS I, and the balance between the maximum activities of Cyt $\hbox {b}_{6}\hbox {f}$ and Rubisco. We focus on how these factors are expressed in observable patterns of gas-exchange and fluorescence. These observables are insensitive to the mechanism of photosynthetic control (i.e., Case 7 or Case 8).

**Fig. 8:**
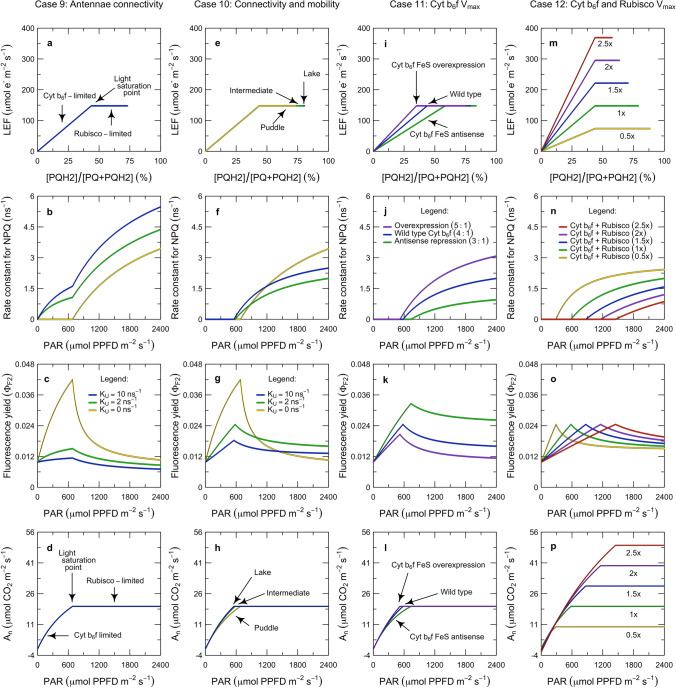
Key model predictions related to the interactions between electron transport and carbon metabolism. These simulations address how the interactions between electron transport and carbon metabolism are expressed in observable fluorescence and gas-exchange parameters. The simulations examine the effects of: the connectivity of the PS II antennae (Case 9), the interaction between the connectivity of the PS II antennae and the redistribution of excitation from PS II to PS I via state transitions (Case 10), the maximum activity of Cyt $\hbox {b}_{6}\hbox {f}$ alone (Case 11), and the interaction between the maximum activities of Cyt $\hbox {b}_{6}\hbox {f}$ and Rubisco (Case 12). Results are plotted in terms of the relationship between $\hbox {PQH}_2$ and LEF, and the light responses of NPQ, the steady-state fluorescence yield of PS II, and net $\hbox {CO}_2$ assimilation. The input parameters are given in Table 2, with one exception: for Cases 11-12, $K_{U2} = 2$ n$\hbox {s}^{-1}$ and state transitions are permitted. Other details are as described in ‘Parameterization of the model (Cases 9–12)’

We begin with the effects of the level of connectivity and mobility of the pigment system (Fig. 8, Cases 9 and 10). The exact mechanistic basis of PS II antennae connectivity, and its magnitude in quantitative terms, are important unknowns because connectivity has the potential to influence both photochemical and non-photochemical quenching. In this initial simulation, we have kept the absorption cross-sections fixed and varied connectivity alone (Fig. 8, Case 9). With this approach, connectivity has no effect on electron transport or $\hbox {CO}_2$ assimilation (Fig. 8a, d). The reason for this is that the development of heat-dissipating NPQ compensates for the excitation subsidy generated by connectivity (Fig. 8b). As a result, the main observable effect of connectivity is on the steady-state fluorescence yield (Fig. 8c). In the next simulation, we have again varied the level of connectivity within the PS II antennae, but have now also permitted the pigment system to dynamically redistribute pigment between PS II and PS I under limiting light intensities (Fig. 8, Case 10). Here, the excitation subsidy generated by connectivity is redistributed from PS II to PS I via state transitions. For puddle-type, intermediate-type, and lake-type connectivity, there are 0%, 17%, and 22% decreases in the PS II cross-section and complementary increases in the PS I cross-section between complete darkness and the light saturation point. Under limiting light intensities, this pigment redistribution enhances the efficiency of electron transport and $\hbox {CO}_2$ fixation (Fig. 8e, h) and eliminates the induction of heat-dissipating forms of NPQ (Fig. 8, f). Under saturating light intensities, the pigment distribution is specified to remain fixed at the value reached at the light saturation point. This dampens the development of heat-dissipating forms of NPQ (Fig. 8f) and enhances the steady-state fluorescence yield (Fig. 8g). Since observed magnitudes of the yields for NPQ (Fig. 2h) and fluorescence (Fig. 2i) are most consistent with the concept that the PS II antennae has a finite level of connectivity which drives pigment redistribution under limiting light, we retain $K_{U2} = 2 \,\hbox {ns}^{-1}$ and permit pigment redistribution for all of the remaining simulations.

The final pair of simulations examines the balance between the maximum activities of Cyt $\hbox {b}_{6}\hbox {f}$ and Rubisco (Fig. 8, Cases 11 and 12). We have simulated the effects of perturbing this ratio with antisense repression and overexpression of Cyt $\hbox {b}_{6}\hbox {f}$ (Fig. 8, Case 11). Decreasing ${V}_{\mathrm{max}}$ for Cyt $\hbox {b}_{6}\hbox {f}$ decreases the LEF that is sustained at a given level of plastoquinone reduction under limiting light (Fig. 8i) and increases the light intensity at which net $\hbox {CO}_2$ assimilation saturates (Fig. 8l). It slightly increases NPQ under limiting light and substantially decreases NPQ under saturating light (Fig. 8j). It also increases the steady-state fluorescence yield under limiting light (Fig. 8k). Overexpression of Cyt $\hbox {b}_{6}\hbox {f}$ has the opposite effects, but the phenotype is relatively more subtle (Fig. 8i–l). We have also simulated the effects of varying ${V}_{\mathrm{max}}$ for Cyt $\hbox {b}_{6}\hbox {f}$ and Rubisco simultaneously, while maintaining a constant ratio between the maximum activities of the two enzymes (Fig. 8, Case 12). This pattern is particularly important from an ecological perspective because it defines the major axis of natural variation in photosynthetic capacity. In these simulations, the redox poise at which the plastoquinone pool transitions from light limitation to light saturation emerges as a constant (Fig. 8m). However, there is a progressive increase in the light intensity at which net $\hbox {CO}_2$ assimilation saturates with the increasing ${V}_{\mathrm{max}}$ values (Fig. 8p). Across the full range of light intensities, the level of NPQ is highest in the simulations with the lowest photosynthetic capacities and vice versa (Fig. 8n). Under limiting light intensities, there is an inverse relationship between photosynthetic capacity and the steady-state fluorescence yield; under saturating light intensities, this pattern is reversed (Fig. 8o). Although the passive emission of fluorescence is a small fraction of the leaf energy budget, the fact that it has a unique relationship with photosynthetic capacity is of particular interest because it is accessible at large spatial scales via proximal and remote sensing.

## Discussion

### Overview

Decades ago, in vitro studies established that the kinetics of plastoquinol oxidation at Cyt $\hbox {b}_{6}\hbox {f}$ are rate-limiting for LEF, and are subject to feedback regulation based on the excitation balance of PS II and PS I as well as the activity of carbon metabolism (West and Wiskich 1968; Murata 1969; Stiehl and Witt 1969). In this paper, we have developed a conceptual and quantitative model that relates these biochemical properties of Cyt $\hbox {b}_{6}\hbox {f}$ to the steady-state dynamics of photosynthesis in intact $\hbox {C}_{3}$ leaves. This model is differentiated from existing models of electron transport in two respects. First, it is capable of simulating many of the characteristic features of gas-exchange and fluorescence in $\hbox {C}_{3}$ leaves based on mechanistic hypotheses about the key complexes and the regulatory interactions between them. This sets it apart from the existing empirical models that achieve comparable or nearly comparable simulation skill (e.g., Farquhar and Wong 1984; Collatz et al. 1991; van der Tol et al. 2014). Second, it has a simple structure because it represents the interactions between electron transport and carbon metabolism and takes advantage of the constraints associated with energy balance, mass balance, and charge balance. This sets it apart from existing mechanistic models that have complex structures because they represent sub-systems and/or transient dynamics and do not take advantage of the same boundary conditions (e.g., Morales et al. 2018b; Bennett et al. 2018; Matuszyńska et al. 2019). In the next sections, we discuss the insights this model provides and the questions it raises about how photosynthesis works, and the potential applications of this framework.

### Limits of electron transport

In quantitative plant physiology, the light response of leaf-level photosynthesis has traditionally been described with empirical functions that quantify some combination of the initial slope, curvature, and asymptote—but leave the underlying mechanisms undefined (e.g., Thornley 1976). The model we have developed here interprets the initial slope and curvature as a consequence of the way that Cyt $\hbox {b}_{6}\hbox {f}$ coordinates electron transport through PS II and PS I under limiting light intensities, and the asymptote as a consequence of the onset of photosynthetic control of Cyt $\hbox {b}_{6}\hbox {f}$ under saturating light intensities. In this section, we begin with a discussion of the limiting light regime, and in the next section turn to the saturating light regime.

#### Excitation balance of PS II and PS I

This model is organized around the hypothesis that the distribution of excitation between PS II and PS I is regulated in such a way as to minimize losses of absorbed light and maximize potential electron transport through Cyt $\hbox {b}_{6}\hbox {f}$ (Fig. 1). The expression for the potential electron transport rate has the form of a Michaelis-Menten expression for a single substrate (i.e., light), but describes the kinetic behavior of the entire electron transport chain (i.e., including both photochemical and biochemical reactions). It predicts that electron transport has a hyperbolic dependence on irradiance, with the maximum efficiency realized at the limit where absorbed irradiance goes to zero and the maximum speed realized at the limit where absorbed irradiance is infinite (Fig. 6, Case 1). The trade-off between the speed and efficiency of potential electron transport is driven by the need for the supplies of reduced plastoquinone and oxidized plastocyanin to be balanced in order to sustain Cyt $\hbox {b}_{6}\hbox {f}$ turnover at the maximum catalytic rate. This causes progressive closure of the PS II and PS I reaction centers, with PS II accumulating in a reduced state and PS I in an oxidized state. As the excitation pressure on PS II and PS I increases, the closure of the reaction centers causes the photochemical yields of PS II and PS I as well as the absorbed quantum yield to decrease as the potential electron flow through Cyt $\hbox {b}_{6}\hbox {f}$ and the potential photosynthetic rate increase.

In general, these modeled patterns are consistent with what is typically observed in assessments of the photosynthetic rate and quantum yield with $\hbox {CO}_2$ and $\hbox {O}_2$ exchange, of the PS II photochemical yield with fluorescence in the range of 650–850 nm, and of the PS I photochemical yield with absorbance in the range of 810-830 nm (e.g., Genty and Harbinson 1996; Baker and Oxborough 2004; Baker et al. 2007; Cornic and Baker 2012; Harbinson and Yin 2017). However, there is currently a substantial imbalance between: (i) the clear and consistent evidence that the distribution of excitation between PS II and PS I is regulated in such a way as to minimize losses of absorbed light, and (ii) the relative paucity of direct quantitative evidence regarding how balancing is achieved in vivo. At present, there is no consensus regarding the nature or extent of PS II connectivity, or how this fits into the overall functioning of photosynthesis (e.g., Mirkovic et al. 2016; Morris and Fleming 2018; Bennett et al. 2019; Oja and Laisk 2020). In this regard, the simulations we have presented are important in that they indicate that the connectivity of PS II may be a previously underappreciated control on the excitation balance of PS II and PS I, and that connectivity-induced excitation imbalances may potentiate state transitions as a re-balancing mechanism (Fig. 8, Cases 9 and 10). On the one hand, the inversion results we have presented are generally consistent with earlier evidence that PS II exhibits an intermediate degree of connectivity (i.e., in between the ‘puddle’- and ‘lake’-type distributions; Kramer et al. 2004; Stirbet 2013) and that state transitions modulate the relative sizes of the PS II and PS I antennae by 10-20% (Kim et al. 2015; Taylor et al. 2019). On the other hand, the fitting exercise certainly does not prove this definitively, and further experimental evaluation is clearly needed.

#### Kinetic bottleneck at Cyt $\hbox {b}_{6}\hbox {f}$

The key prediction of the expression for the potential electron transport rate is that the maximum activity of Cyt $\hbox {b}_{6}\hbox {f}$ defines the upper limit for the theoretical maximum speed of electron transport. This prediction can be tested in several ways. One is by changing the maximum activity of Cyt $\hbox {b}_{6}\hbox {f}$ in the model, and qualitatively comparing the model’s response to observations. The model predicts that increases in Cyt $\hbox {b}_{6}\hbox {f}$ concentration cause increases in the potential rates of electron transport and $\hbox {CO}_2$ assimilation at a given light intensity, and vice versa (Fig. 6, Case 2; Fig. 8, Case 11 and Case 12). A positive correlation between Cyt $\hbox {b}_{6}\hbox {f}$ content and leaf assimilation capacity has been observed consistently in studies of light acclimation, with the highest Cyt $\hbox {b}_{6}\hbox {f}$ contents and leaf assimilation capacities associated with the highest light growth conditions (Björkman et al. 1972; Evans 1987; Anderson 1992; Yamori et al. 2010; Schöttler and Tóth 2014). A second way of testing the prediction is to quantitatively compare modeled and measured maximum activities of Cyt $\hbox {b}_{6}\hbox {f}$. The inversion provides an estimate that the ratio between the maximum activities of Cyt $\hbox {b}_{6}\hbox {f}$ and Rubisco was about 3.4 (3.0, 3.9) mol $\hbox {e}^{-}$ $\hbox {mol}^{-1}$ $\hbox {CO}_2$ for the *P. fremontii* (Fig. 5). This is close to, but slightly lower than, the range of 3.7 to 5.0 mol $\hbox {e}^{-}\,\hbox {mol}^{-1}\,\hbox {CO}_2$ found for *Nicotiana tabacum* (Yamori et al. 2010). The difference could reflect the lower maximum growth light intensity for *N. tabacum* versus *P. fremontii* (i.e., 450 vs. $800\,\upmu$mol PPFD $\hbox {m}^{-2}\,\hbox {s}^{-1}$) and/or statistical errors in the parameter estimation procedure. A third way of testing the prediction is to examine the effects of genetic manipulations of the maximum activity of Cyt $\hbox {b}_{6}\hbox {f}$. Decreases in the light-limited rates of electron transport $\hbox {CO}_2$ assimilation have been observed consistently in studies in which the Cyt $\hbox {b}_{6}\hbox {f}$ Rieske FeS protein was suppressed through transgenic techniques (Price et al. 1995; Hurry et al. 1996; Anderson et al. 1997; Price et al. 1998; Eichelmann and Laisk 2000; Ruuska et al. 2000; Eichelmann et al. 2009; Yamori et al. 2011, 2016). Increases in the light-limited rates of electron transport and $\hbox {CO}_2$ assimilation have also been observed in recent work in which the Cyt $\hbox {b}_{6}\hbox {f}$ Rieske FeS protein was overexpressed through transgenic techniques, albeit with a phenotype that is more subtle than the antisense phenotype (Simkin et al. 2017; Ermakova et al. 2019). All of these observations are consistent with the Cyt $\hbox {b}_{6}\hbox {f}$-based expression we have described for potential electron transport. One of the interesting features of this comparison is that the model can provide a completely specific manipulation of Cyt $\hbox {b}_{6}\hbox {f}$. While single gene manipulations are often conceptualized as equivalently targeted perturbations, genetic changes can be translated into the phenotype in much more complex ways. This highlights the utility of a quantitative framework for interpreting the relationships between genotype, phenotype, and performance.

#### Coupling efficiency

To simulate maximal efficiency of coupling between electron flow and the proton circuit, we have combined the assumptions that the Cyt $\hbox {b}_{6}\hbox {f}$ Q-cycle operates constitutively in LEF as well as CEF1 (Sacksteder et al. 2000), the ATP synthase operates at the thermodynamic stoichiometry rather than the structural stoichiometry (Petersen et al. 2012), and the NDH complex functions as a high efficiency proton pump in CEF1 (Strand et al. 2017). With this approach, the flux through CEF1 only needs to be a few percent of the flux through LEF in order to satisfy the energetic demands of the PCR and PCO cycles, and any relaxation of the assumptions about coupling efficiency increases the partitioning to CEF1 (Fig. 6, Case 3). These results are consistent with the interpretation that in $\hbox {C}_{3}$ plants LEF is the dominant pathway for electron flow (Baker et al. 2007; Cornic and Baker 2012), but some form of CEF1 plays an essential role in balancing the reductant and ATP budgets (Yamori and Shikanai 2016; Nawrocki et al. 2019). Since the molecular details of the CEF1 pathway(s) continue to be a source of debate, it is also useful to separate the assumptions about CEF1 from those about Cyt $\hbox {b}_{6}\hbox {f}$ and ATP synthase by using elevated $\hbox {CO}_2$ to suppress PCO cycle activity. Under this condition, the modeled values of the maximum absorbed quantum yield are in good agreement with measured values (e.g., compare Fig. 6, Case 4, with Björkman and Demmig 1987; Long et al. 1993; Hogewoning et al. 2012). These results are consistent with the operation of the Cyt $\hbox {b}_{6}\hbox {f}$ Q-cycle and operation of the ATP synthase at the thermodynamic stoichiometry at the limit where light goes to zero. While we have specified in these simulations that the coupling efficiency remains constant under all limiting light intensities, and while this assumption is also applied in the inversion, it is difficult to evaluate this unambiguously with gas-exchange and fluorescence measurements. There are a number of mechanisms that could cause the coupling efficiency to decrease as the proton motive force builds up, such as a decreased rate of proton pumping into the lumen or an increased rate of proton leakage out of the lumen. If dynamic changes in coupling efficiency are a feature of steady-state photosynthesis, then their omission from the steady-state model will result in errors that alias onto the free variables in the fitting procedure (Fig. 5). This is both a challenge and an opportunity. To study the coupling efficiency quantitatively in vivo, the fitting approach can be expanded to include paired biochemical measurements as well as absorbance-based measurements that probe PS I (810-830 nm), the electrochromic shift (500-540 nm), and Cyt $\hbox {b}_{6}\hbox {f}$ (540-570 nm) (e.g., Hall et al. 2013).

### Regulation of electron transport

Considering that the significance of the kinetic bottleneck at Cyt $\hbox {b}_{6}\hbox {f}$ has long been appreciated in qualitative terms, some discussion is needed of the reasons why this phenomenon has been missing a clear quantitative expression. In our view, the major problem has been that the $J_{\max }$-based expressions for potential electron transport did not differentiate clearly between the conditions where Cyt $\hbox {b}_{6}\hbox {f}$ activity exerts control over carbon metabolism versus where carbon metabolism exerts control over Cyt $\hbox {b}_{6}\hbox {f}$ activity. Conflating these two different regulatory domains in the $J_{\max }$-based expressions inspired attempts to assay the maximum capacity for electron transport in vivo using saturating irradiance and saturating $\hbox {CO}_2$. However, these conditions elicit a state in which the $\hbox {PQH}_2$ pool is only partially reduced and Cyt $\hbox {b}_{6}\hbox {f}$ is operating under the control of feedback from downstream reactions (e.g., see discussion by Laisk et al. 2005b). As a result, the derived rates of electron flow per unit Cyt $\hbox {b}_{6}\hbox {f}$ (e.g., 110–160  $\hbox {s}^{-1}$; Niinemets and Tenhunen 1997; Yamori et al. 2010) have far underestimated the potential rates of electron flow per unit Cyt $\hbox {b}_{6}\hbox {f}$ (e.g., 290–450 $\hbox {s}^{-1}$ at 25 $^{\circ }\hbox {C}$; Dietrich and Kühlbrandt 1999; Zhang et al. 2001). This discrepancy is reconciled by the model we have described here. The key is recognizing that there are two regimes for electron flow through Cyt $\hbox {b}_{6}\hbox {f}$: under limiting light, the rate is determined by the interaction between the $\hbox {PQH}_2$ supply and the maximum turnover constant of the enzyme, whereas under saturating light the rate is determined by the interaction between the $\hbox {PQH}_2$ supply and a downregulated turnover rate (Figs. 3, 4, and 7). In this section, we turn to consideration of the regulatory processes that coordinate electron transport with carbon metabolism.

#### Photosynthetic control

The expressions describing feedback control over Cyt $\hbox {b}_{6}\hbox {f}$ activity are based on the idea that Cyt $\hbox {b}_{6}\hbox {f}$ functions like a transistor, i.e., a component of an electrical circuit that uses variable conductance to control current (Fig. 1; blue transistor and photosynthetic control arrow). Expressed via analogy to Ohm’s law, Cyt $\hbox {b}_{6}\hbox {f}$ presents a constant, maximal conductance under light-limiting conditions (which maximizes LEF to the sink), and a lower, variable conductance under light-saturating conditions (which minimizes LEF in excess of the capacity of the sink). With experimental data, the conductance of Cyt $\hbox {b}_{6}\hbox {f}$ can be estimated as $k_{Puddle} = LEF/(1 - qP)$ or $k_{Lake} = LEF/(1 - qL)$, depending on whether the apparent redox state of the PQ pool is given by $1 - qP$ (Schreiber et al. 1986) or $1 - qL$ (Kramer et al. 2004). Both indices provide evidence that the conductance of Cyt $\hbox {b}_{6}\hbox {f}$ is subject to regulation at the dark-light transition as well as at the transition from limiting to saturating light (Fig. 5i, k). However, they also give quite different estimates of the conductance of Cyt $\hbox {b}_{6}\hbox {f}$ within the limiting light range: $k_{Puddle}$ is relatively high and appears to decline continuously, whereas $k_{Lake}$ is relatively low and appears to increase continuously. The interpretation that is most consistent with our analyses is that the true connectivity of the PS II antennae is intermediate (i.e., between puddle- and lake-type configurations, but closer to the latter) and that the true conductance of Cyt $\hbox {b}_{6}\hbox {f}$ is constant under limiting light (i.e., between $k_{Puddle}$ and $k_{Lake}$, but again closer to the latter).

The fact that Cyt $\hbox {b}_{6}\hbox {f}$ can modulate its conductance to LEF within milliseconds of a perturbation in light suggests that photosynthetic control is the first line of defense against overexcitation, protecting the acceptor side of PS I from being flooded with highly reduced intermediates (Fig. 4c). At present, it is not clear whether this response involves exactly the same mechanisms on the flash and steady-state timescales. The classic concept has been that photosynthetic control of Cyt $\hbox {b}_{6}\hbox {f}$ is potentiated by acidification of the bulk lumenal proton pool, which exerts backpressure on the proton-coupled electron flow from $\hbox {PQH}_2$ to ${\hbox {PC}_{\mathrm{ox}}}$ (West and Wiskich 1968). However, as discussed in ‘Experiment’, previous in vivo studies have not yielded clear evidence that this is the mechanism that is normally engaged at the light saturation point. Further, this mechanism seems likely to be much too slow to mediate a feedback that can be activated within tens of milliseconds, as the flash analysis suggests. In this context, the simulations delineate two broad ways that photosynthetic control could potentially work in the steady-state: a decrease in the absolute value of the rate constant for $\hbox {PQH}_2$ oxidation at Cyt $\hbox {b}_{6}\hbox {f}$ (Fig. 7, Case 7), or a decrease in the apparent value of this rate constant due to increased competition from electrons in a regulatory form of CEF1 (Fig. 7, Case 8). Depending on how Cyt $\hbox {b}_{6}\hbox {f}$ works, one effect might predominate, or both might be partially expressed at the same time.

While most of the details of how photosynthetic control works are open questions, the sine wave experiment provides insight about how photosynthetic control fits into the overall regulation of photosynthesis. In response to a sustained increase in light, the induction of photosynthetic control is followed by induction of NPQ; as NPQ alleviates the electron overpressure in the PQ pool, photosynthetic control progressively relaxes; and the two forms of regulation gradually settle to a steady-state at the new light intensity (Figs. 3, 4). This interaction seems to allow electron transport to proceed at the Cyt $\hbox {b}_{6}\hbox {f}$-limited rate under low light intensities, and then smoothly switch to the Rubisco-limited rate once the light intensity is high enough to become saturating (Fig. 7, Cases 7 and 8). It also seems to allow photosynthesis to operate safely and efficiently in a wide range of biochemical milieus, from those characteristic of natural variation in photosynthetic capacity (with balanced electron transport and carbon metabolism) to those characteristic of genetic manipulations (with imbalances in electron transport and carbon metabolism) (Fig. 8, Cases 11 and 12). The concept that the control of the speed of intersystem electron transport fundamentally resides in Cyt $\hbox {b}_{6}\hbox {f}$ opens a new perspective on the function of NPQ.

#### Non-photochemical quenching

The conventional interpretation is that the family of NPQ processes has the function of directly protecting PS II from damage (by dissipating excess excitation from the antennae) and indirectly protecting PS I from damage (by limiting electron transfer through Cyt $\hbox {b}_{6}\hbox {f}$) (e.g., Demmig-Adams et al. 2014). However, a purely photoprotective function is difficult to reconcile with three observations. First, a significant fraction of NPQ develops under limiting light where no excess excitation is expected, there is no abrupt change in NPQ at the light saturation point, and the level of NPQ that develops under saturating light does not prevent the PQ pool from continuing to become reduced (Figs. 2, 3, and 5). Second, the suppression of NPQ by the chemical inhibitor dithiothreitol is not associated with any inhibition of PS II photochemistry, but only with increased closure of PS II reaction centers (e.g., Bilger and Björkman 1990). Third, mutants like *npq1* and *npq4* with suppression of NPQ generally exhibit over-reduction of the PQ/$\hbox {PQH}_2$ pool, but do not lose control of intersystem electron transport (Niyogi et al. 1998) or suffer from photodamage of PS I (e.g., Tikkanen et al. 2015). These observations suggest that: (i) NPQ has a function that is distinct from, but complementary to, photoprotection; and (ii) the key to understanding this function is defining ‘excess excitation’ quantitatively and within the context of the overall photosynthetic system.

The model we have described here provides just such a definition (Fig. 1; non-photochemical quenching arrow). The model simulations indicate that NPQ does not need to be engaged when the pigment distribution is fixed in such a way as to exactly balance the excitation of PS I and PS II (Fig. 7, Case 5). The model simulations also indicate that the excitation of PS II and PS I is susceptible to imbalances that are related to the absorbance cross-section of each population of photosynthetic units and the connectivity between photosynthetic units, and that either energy-conserving or energy-dissipating forms of NPQ can be engaged to correct such imbalances (Fig. 8, Cases 9 and 10). These dynamics are consistent with the interpretation that the family of NPQ processes functions to control the excitation balance of PS II and PS I in relation to the demand for linear and cyclic electron flow. While the importance of excitation balancing is well-established in qualitative terms, it is less well-defined in quantitative terms. In the model, the optimal excitation balance is represented as one which poises the intersystem chain for maximum electron flow through Cyt $\hbox {b}_{6}\hbox {f}$ (Eqs. 28 and 34). The model inversion provides a test of the explanatory power of this idea (Fig. 5).

In the inversion, the model is configured to permit state transitions to dynamically optimize the distribution of absorbed light under limiting light intensities, such that the closure of PS II and PS I is exactly balanced and the accumulation of reduced plastoquinone is as high as possible at a given light intensity. The model is also configured to hold the pigment distribution constant once the light saturation point is reached, and to induce heat-dissipating NPQ to a level that continues to poise the plastoquinone pool to maximize potential electron flow through Cyt $\hbox {b}_{6}\hbox {f}$. In general, the inversion results support these concepts because they indicate good agreement between the modeled and measured values of the yield for NPQ ($\varPhi _{N2}$; Fig. 5c, d) as well as the reduction of the plastoquinone pool (either estimated as $1-qP$ or $1-qL$; Fig. 5e–h). However, the inversion results also indicate: (i) that heat-dissipating forms of NPQ start to develop under limiting light intensities, i.e, significantly before the light saturation point; and (ii) that at all light intensities, the need for heat-dissipating NPQ depends quantitatively on the distribution of antennae pigments between PS II and PS I. These results raise the question of whether and if so, how, cyclic electron flow around PS I interacts with non-photochemical quenching and photosynthetic control during transitions between the Cyt $\hbox {b}_{6}\hbox {f}$-limited and Rubisco-limited states.

#### Cyclic electron flow

The potential role(s) of CEF1 in photosynthetic control have been much discussed in relation to the phenotypes of the *pgrl1* and *pgr5* mutants, and this model offers a new quantitative lens on this problem (e.g., Munekage et al. 2001, 2002, and subsequent studies). Since the gas-exchange and fluorescence measurements we made in this study do not provide direct constraints on CEF1, we formulated this model to represent the lower and upper bounds on CEF1: the minimum potential CEF1 that would be required by the ATP demands of carbon metabolism, and the maximum potential CEF1 that could be driven by the PS I excitation in excess of the demands of carbon metabolism (Fig. 1; cyclic electron flow). These bounds lead to three questions about how regulation negotiates the transition from limiting to saturating light. First, is the downregulation of Cyt $\hbox {b}_{6}\hbox {f}$ turnover under saturating light real, or is it only an apparent downregulation due to increased CEF1? Second, why does the PQ/$\hbox {PQH}_2$ pool appear to be poised for maximum flow through Cyt $\hbox {b}_{6}\hbox {f}$ under limiting as well as saturating light? Third, why is heat-dissipating NPQ induced before the light saturation point?

If the downregulation of Cyt $\hbox {b}_{6}\hbox {f}$ turnover under saturating light reflects a slowing of $\hbox {PQH}_2$ oxidation at Cyt $\hbox {b}_{6}\hbox {f}$, this suggests an analogy in which Cyt $\hbox {b}_{6}\hbox {f}$ functions like a field effect transistor. In this type of transistor, the application of a voltage to a ‘gate’ terminal controls the flow of current between ‘source’ and ‘drain’ terminals. There is some empirical support for such a scenario from in vivo measurements of Cyt f reduction which indicate slowing of the effective rate constant for $\hbox {PQH}_2$ oxidation under saturating light and low $\hbox {CO}_2$ (i.e., based on the absorbance change at 554 nm during rapid light-dark transitions; Takizawa et al. 2007). However, in this scenario it is not intuitively obvious how to interpret the induction of heat-dissipating NPQ under limiting light or the steady-state poise of the PQ/$\hbox {PQH}_2$ pool under saturating light. One possibility is that both features function to balance photosynthetic efficiency and safety as light availability fluctuates. Since the modulation of NPQ occurs more slowly than the modulation of Cyt $\hbox {b}_{6}\hbox {f}$ turnover, NPQ could be interpreted as a control which maximizes potential LEF and CEF1 in the event that acceptors are available, and photosynthetic control could be interpreted as a control which restricts actual LEF and CEF1 when acceptors are limited (Fig. 7, Case 7).

Alternatively, there might be no downregulation of Cyt $\hbox {b}_{6}\hbox {f}$ turnover under saturating light if the control of LEF is mediated by CEF1. This suggests an analogy in which Cyt $\hbox {b}_{6}\hbox {f}$ functions like a bipolar junction transistor. In this type of transistor, the application of a small current to a ‘base’ terminal controls the flow of a larger current between ‘emitter’ and ‘collector’ terminals. There is also some empirical support for this scenario (e.g., Miyake et al. 2005; Laisk et al. 2007). On the one hand, this would open questions about the coupling between electron transport and the proton circuit because a regulatory CEF1 flux would need to be accommodated by some type of decoupling mechanism(s), such as disengagement of the Cyt $\hbox {b}_{6}\hbox {f}$ Q-cycle (e.g., Laisk et al. 2010). On the other hand, it could simplify interpretation of the steady-state poise of the PQ/$\hbox {PQH}_2$ pool under saturating light and the induction of heat-dissipating NPQ under limiting light. Namely, if CEF1 functions as a mechanism of photosynthetic control that maintains total electron flow through Cyt $\hbox {b}_{6}\hbox {f}$ while restricting LEF to acceptor availability, then the induction of heat-dissipating NPQ before the light saturation point could simply permit that CEF1 flux to start up, and the $\hbox {PQH}_2$ poise under saturating light could simply be required to sustain the maximum potential rate of electron transport through Cyt $\hbox {b}_{6}\hbox {f}$ (Fig. 7, Case 8).

### Model development and potential applications

There are a number of opportunities for further development of this model. First, it is likely to be useful to extend the model we have described here to resolve acceptor-side closure of Photosystem I. We have started here with a description of donor-side closure alone because acceptor-side closure is thought to comprise a small fraction of total closure ($<10\%$), and technically has been somewhat difficult to assess (Baker et al. 2007). However, the state of the acceptor side of PS I is central to the supply-demand balance of the photosynthetic system, likely plays a key role in regulatory interactions, and may be more accessible with new measurement techniques (e.g., see Klughammer and Schreiber 2016). Second, it is also likely to be useful to extend the approach to modeling the proton circuit with the $n_L$ and $n_C$ parameters to explicitly resolve the proton motive force and the conductance of the ATP synthase (e.g., see Kanazawa et al. 2017). Third, both of these developments could support a more mechanistic analysis of the temperature responses of photosynthesis, and particularly the temperature responses of electron transport (e.g., see Kruse et al. 2016). Such developments can be efficiently pursued in an inversion framework, as below.

#### Leaf-level applications

At the leaf-level, this model can be used in a forward mode to design experiments, or in an inverse mode to interpret observations—analogous to the ways that the Farquhar et al. (1980) model has been applied (e.g., see reviews by von Caemmerer et al. 2009; von Caemmerer 2013, 2020). In the type of inversion framework we have demonstrated here, the model assumptions can be confronted in a rigorous and reproducible way with gas-exchange, PAM fluorescence, and/or spectrally resolved fluorescence measurements. This provides a new way to analyze the contributions of PS I and PS II to active and passive fluorescence measurements (e.g., Franck et al. 2002; Pfündel et al. 2013). However, the inversion approach is likely to be much more powerful with the inclusion of absorbance-based measurements that directly probe PS I (810-830 nm), the electrochromic shift (500-540 nm), and Cyt $\hbox {b}_{6}\hbox {f}$ (540-570 nm) (e.g., Laisk et al. 2002; Hall et al. 2013; Klughammer and Schreiber 2016). The model can be extended to simulate each of these signals. With these elements, an inversion-based approach can be used to quantitatively relate the physiological fluxes to the biochemical and anatomical properties of leaves.

#### Applications across other scales of organization

This model is also suitable for several types of applications at other scales. First, it can be used to establish boundary conditions for the more detailed mechanistic models that are being developed for studies of sub-systems and transients (e.g., Morales et al. 2018b; Bennett et al. 2018; Matuszyńska et al. 2019, and related models). This approach may be useful in interpreting the properties of mutant or genetically engineered plants, and should lead to a more complete understanding of the molecular basis of photosynthetic responses to the environment. Second, it can be used to replace the empirical models of electron transport and NPQ that are currently relied on for representation of leaf-level processes in canopy-level modeling frameworks (e.g., Farquhar and Wong 1984; Collatz et al. 1991; van der Tol et al. 2014, and related models). This should provide an improved basis for interpreting measurements of solar-induced chlorophyll fluorescence from proximal and remote sensing, and open new opportunities for simulating canopy-level photosynthesis in the land surface component of weather and climate models.

## Conclusions

1. We have developed new experimental methods which use PAM fluorescence measurements to estimate the maximum activity of Cyt $\hbox {b}_{6}\hbox {f}$ in vivo, and to identify the conditions under which feedback control of Cyt $\hbox {b}_{6}\hbox {f}$ is active or relaxed. The application of these methods to the analysis of the photosynthetic light response reveals two features of the regulation of electron transport. First, the continuous curvature of the light response is caused by the kinetic restriction that Cyt b_6_f presents to electron flow through PS II and PS I. Second, photosynthetic control of Cyt b_6_f can slow LEF within a few milliseconds of a perturbation in light, much more quickly than NPQ.
2. Based on these observations, we have formulated a new model of electron transport that is coupled to the model of carbon metabolism introduced by Farquhar et al. (1980). The model is based on quantification of the energy and mass flows linking PS I and PS II and their associated pigment systems with Cyt $\hbox {b}_{6}\hbox {f}$, the ATP synthase, and Rubisco. It resolves the demand for energy from the PCR and PCO cycles, the supply of energy from LEF and CEF1, and how the supply/demand balance relates to the partitioning of absorbed light between photochemistry, heat dissipation, and fluorescence. This simplified structure permits one to analyze how the rate-limiting steps and their regulation determine the environmental responses of the intact system.
3. In this framework, the excitation balance of PS II and PS I and the maximum activities of Cyt $\hbox {b}_{6}\hbox {f}$ and Rubisco emerge as key limits on system dynamics. For example, the trade-off between the speed and efficiency of electron transport is shown to be controlled by the excitation balance of PS II and PS I and the maximum activity of Cyt $\hbox {b}_{6}\hbox {f}$. The development of NPQ is shown to be controlled by the excitation balance of PS II and PS I and the demand for LEF and CEF1. The onset of photosynthetic control is shown to be dependent on the maximum activities of Cyt $\hbox {b}_{6}\hbox {f}$ and Rubisco.
4. This framework has a range of potential applications in analyzing and simulating photosynthesis. While this paper focuses on the light response of leaves, the model is fully capable of simulating responses of leaf photosynthesis to $\hbox {CO}_2$, $\hbox {O}_2$, and temperature using parameterizations already developed for the Farquhar et al. (1980) model. Therefore, this model can be substituted for the Farquhar et al. (1980) model in any application now using it. This provides an opportunity to explore one of the mysteries of photosynthesis in higher plants—how PS I and PS II work together.

## Data Availability

The version of the model described in this manuscript has been implemented in MATLAB R2020b and is compatible with GNU Octave 5.2.0. The code is published under an MIT License and archived on Zenodo (10.5281/zenodo.4759246).

## Abbreviations

ATP synthase: Chloroplastic ATP synthase
CEF1: Cyclic electron flow around PS I
Cyt $\hbox {b}_{6}\hbox {f}$: Cytochrome $\hbox {b}_{6}\hbox {f}$ complex
$${\hbox {Fd}_{\mathrm{ox}}}$$: Ferredoxin, oxidized
$${\hbox {Fd}_{{\mathrm{red}}}}$$: Ferredoxin, reduced
LEF: Linear electron flow
NPQ: Non-photochemical quenching
PAR: Photosynthetically active radiation
$${\hbox {PC}_{\mathrm{ox}}}$$: Plastocyanin, oxidized
$${\hbox {PC}_{{\mathrm{red}}}}$$: Plastocyanin, reduced
PCO: Photosynthetic carbon oxidation
PCR: Photosynthetic carbon reduction
PPFD: Photosynthetic photon flux density
PQ: Plastoquinone
$${\hbox {PQH}_2}$$: Plastoquinol
PS I: Photosystem I
PS II: Photosystem II
RuBP: Ribulose-1,5-bisphosphate
Rubisco: RuBP carboxylase–oxygenase
